# Energetic stress in combination with impaired fatty acid oxidation induces sequestration of CoA and adaptation of CoA metabolism

**DOI:** 10.1111/febs.70442

**Published:** 2026-02-07

**Authors:** Ligia Akemi Kiyuna, Christoff Odendaal, Madhulika Singh, Albert Gerding, Miriam Langelaar‐Makkinje, Marianne van der Zwaag, Asmara Drachman, Vladimíra Cetkovská, Gaby Liem Foeng Kioen, Anne‐Claire M. F. Martines, Nicolette C. A. Huijkman, Hein Schepers, Bart van de Sluis, Dirk‐Jan Reijngoud, Ody C. M. Sibon, Amy C. Harms, Thomas Hankemeier, Barbara M. Bakker

**Affiliations:** ^1^ Laboratory of Paediatrics, University of Groningen University Medical Centre Groningen The Netherlands; ^2^ M Leiden Academic Centre for Drug Research, Leiden Academic Centre for Drug Research, Leiden University The Netherlands; ^3^ Department of Laboratory Medicine University of Groningen, University Medical Centre Groningen The Netherlands; ^4^ Biomedical Sciences/ERIBA, University Medical Centre Groningen University of Groningen The Netherlands; ^5^ United for Metabolic Diseases (UMD) Groningen The Netherlands

**Keywords:** CASTOR, CoA metabolism, inborn errors of metabolism, MCAD deficiency, systems medicine

## Abstract

Coenzyme A (CoA) is a vital cofactor involved in 8–10% of all metabolic reactions in human cells. Different inherited enzyme deficiencies in which the oxidation of acyl‐CoAs is hampered have been hypothesised to share a phenotype characterised by toxic accumulation of acyl‐CoA and a concomitant decline in free CoA (CoASH) levels, whereby CoASH becomes limiting for other metabolic reactions. This is referred to as CoASH sequestration. There is, however, limited experimental evidence for this hypothesis. Using a combination of approaches, we test this hypothesis in medium‐chain acyl‐CoA dehydrogenase deficiency (MCADD), the most common deficiency of mitochondrial fatty acid oxidation (mFAO), under energetic stress. Both *in vitro* MCAD‐knockout (KO) HepG2 cells and a kinetic model of mFAO showed decreased CoASH, elevated medium‐chain acyl‐CoA, and decreased long‐chain acyl‐CoA levels. MCAD‐KO mice exposed to fasting and cold as energetic stressors had a significantly increased total CoA pool and increased expression of CoA biosynthetic enzymes in the liver, indicative of an upregulated CoA biosynthesis. Expression of carnitine acyltransferases and acyl‐CoA thioesterases, enzymes that liberate CoASH from acyl‐CoAs, was also upregulated, suggesting an adaptive response of CoA metabolism to decreased CoASH. Finally, computational model simulations showed that a combination of elevated total CoA and thioesterase activity led to normalisation of both CoASH and medium‐chain acyl‐CoA levels. Together, the results provide the first evidence for the CoA sequestration hypothesis in MCADD. The observed adaptation of CoA metabolism under energetic stress may act as a compensatory response that counteracts CoASH depletion and accumulation of toxic medium‐chain acyl‐CoAs.

## Graphical abstract

Computational modelling and *in vitro* liver cell experiments indicate that medium‐chain acyl‐CoA dehydrogenase (MCAD) deficiency causes an accumulation of (especially medium‐chain) acyl‐CoAs at the cost of free CoA (CoASH). A substantial decrease in CoASH impairs flux through many pathways essential for energy homeostasis. MCAD‐knockout mice, in response, upregulate CoASH production and acyl‐CoA‐cleaving pathways.

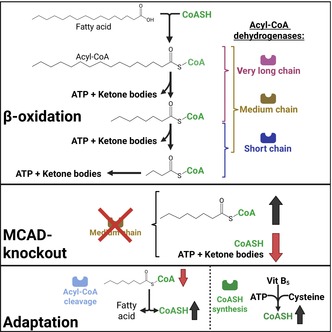

**Abbreviations**
ABCD: ATP‐binding cassette domain transporter
ACOT: acyl‐CoA thioesterase
CASTOR: CoA sequestration, toxicity, and redistribution
CoA: coenzyme A
CoASH: free (non‐esterified) CoA
CoASY: bifunctional Coenzyme A synthetase
CPT: carnitine palmitoyltransferase
CrAT: carnitine acetyltransferase
dPCoA: dephospho‐CoA
FFA: free fatty acid
MCAD: medium‐chain acyl‐CoA dehydrogenase
MCADD: MCAD deficiency
mFAO: mitochondrial fatty acid oxidation
NUDT: nudix hydrolases
PANK: pantothenate kinase
pFAO: peroxisomal fatty acid oxidation
P‐pant: 4′‐phosphopantetheine
PPCDC: phosphopantothenoylcysteine decarboxylase
PPCS: phosphopantothenoylcysteine synthetase
P‐Vit B_5_
: 4′‐phosphopantothenate
P‐Vit B_5_‐Cys: 4′‐phosphopantothenoylcysteine
Vit B_5_
: Vitamin B_5_/pantothenate

## Introduction

Coenzyme A (CoA) is vital for many metabolic pathways, particularly in the mitochondria. The genome‐scale reconstruction of human metabolism, Human1, contains 1044 reactions that sequester or release CoA that is 8% of all reactions included [1], making it one of the most connecting metabolites on the metabolic map. Similarly, of the 19 313 reactions in the Recon3D reconstruction, 1941 reactions (10%), sequester or release CoA [2]. CoA is essential for, among others, the mitochondrial fatty acid oxidation (mFAO) [3], the oxidation of branched‐chain amino acids [4], and the tricarboxylic acid cycle [5]. Classically, symptoms of a metabolic disease are attributed to the function of the pathway in which the defect is found. For instance, an impairment of the mFAO limits the availability of energy from fat. It has been hypothesised, however, that a range of diseases in which CoA is implicated, might also exert their pathogenicity via the accumulation of CoA esters and the concomitant depletion of the free form of CoA (CoA molecules not esterified to carbon chains; free CoA is abbreviated as CoASH). This hypothesis is called CASTOR: Coenzyme A Sequestration, Toxicity, and Redistribution [6, 7, 8]. Common symptoms of CASTOR diseases include acidosis, hypoglycaemia and hyperammonaemia, often with liver, heart, or multiple organ dysfunction. These common symptoms are attributed to a shortage of CoASH, which is required for gluconeogenesis and oxidative phosphorylation, as well as indirectly to a lack of acetyl‐CoA for ureagenesis [6, 9].

**Fig. 1:**
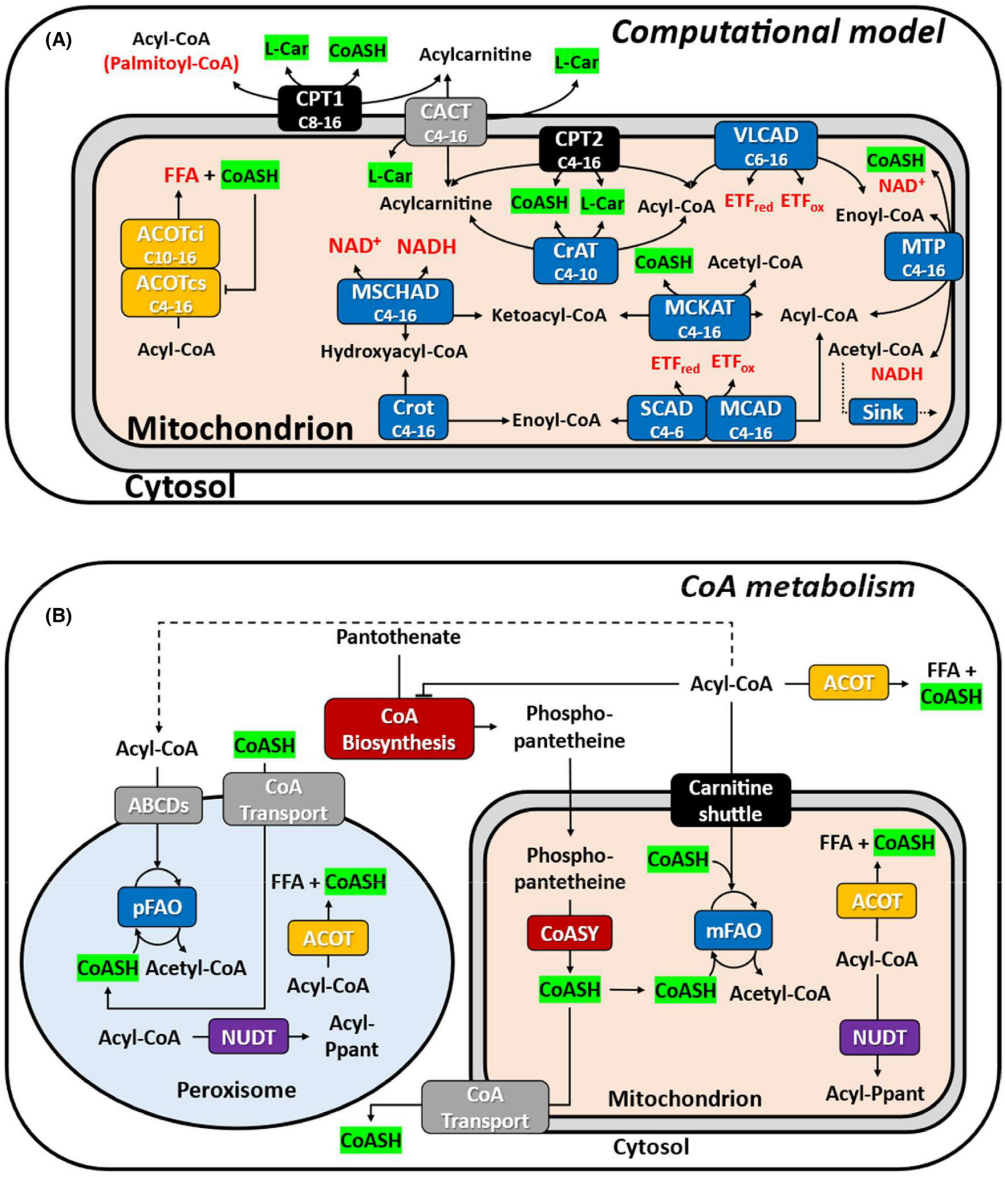
Coenzyme A compartmentalisation, synthesis, and sequestration. Boxes indicate pathways or individual enzymes: carnitine acyltransferases in black, transporters in grey, thioesterases in yellow, nudix hydrolases in purple, fatty acid oxidation in blue and CoA (coenzyme A) biosynthesis in red. Black text refers to metabolites. CoASH and L‐Car (highlighted green) represent free CoA and L‐carnitine, respectively. (A) Reactions included in the kinetic computational model of human liver mitochondrial fatty acid oxidation (mFAO). Red metabolites have fixed concentrations in the model. The chain‐length specificity of each of the reactions is indicated in small font below the enzyme name (e.g. C8–C16). ACOTci/cs, CoASH‐insensitive/‐sensitive ACOT; ETFred/ox, reduced/oxidised electron‐transferring flavoprotein; CPT1/2, carnitine palmitoyltransferase 1/2; CACT, carnitine acylcarnitine translocase; CrAT, carnitine acetyltransferase; Crot, crotonase; MTP, mitochondrial trifunctional protein; MSCHAD, medium‐ and short‐chain hydroxyacyl‐CoA dehydrogenase; MCKAT, medium‐chain ketoacyl‐CoA thiolase; VLCAD/MCAD/SCAD, very long‐/medium‐/short‐chain acyl‐CoA dehydrogenase. (B) Pathways investigated in this study span three compartments: the cytosol, mitochondrion, and peroxisome. ABCD, ATP‐binding cassette domain transporter; CoASY, bifunctional CoA synthase; FFA, free fatty acid; NUDT, nudix hydrolase (nucleoside diphosphate linked moiety X‐type motif); pFAO, peroxisomal fatty acid oxidation.

Medium‐chain acyl‐CoA dehydrogenase deficiency (MCADD, #OMIM 201450) is the most prevalent inborn error of the mitochondrial fatty acid oxidation (9). In humans, the medium‐chain acyl‐CoA dehydrogenase (MCAD) enzyme catalyses the conversion of acyl‐CoA esters with a chain length of 6–12 carbons into the corresponding enoyl‐CoA esters [10] (Fig. 1A). It is considered the most important enzyme for the oxidation of medium‐chain fatty acids. Around 80% of symptomatic patients are homozygous for the c.985A>G missense mutation in the *ACADM* gene, with less than 1% residual MCAD activity [11, 12]. In the blood and serum of patients, elevated levels of acylcarnitine esters with acyl chains of 6–10 carbon atoms are observed, as well as an elevated ratio of C8/C10 acylcarnitines [11]. These are thought to reflect elevated levels of the corresponding acyl‐CoA esters in the liver [13, 14]. Before MCADD was widely included in newborn screening programmes [15, 16, 17, 18], it was either detected after the development of symptoms or via proband follow‐up due to a symptomatic sibling [19, 20, 21]. Symptoms mostly occur during the first 5 years of life and involve severe hypoketotic hypoglycaemias in response to energetic stress, such as fasting, cold exposure and intercurrent illness [22]. This hypoglycaemia, in combination with the accumulation of medium‐chain acyl‐CoA esters and hyperammonaemia, would make MCADD a typical CASTOR disease [6].

Direct evidence for the accumulation of CoA esters and depletion of CoASH in putative CASTOR diseases is limited [7, 8, 23]. Most often the cognate carnitine esters are measured [7, 8]. In a mouse model of propionyl‐CoA carboxylase (PCC) deficiency, or propionic acidaemia, however, direct measurement showed concentrations of propionyl‐CoA to be elevated and those of CoASH to be decreased [24, 25]. Like other CASTOR diseases, propionic acidaemia is associated with metabolic acidosis, hypoglycaemia, and hyperammonaemia [26]. In PCC‐deficient mice, pharmacological activation of CoA biosynthesis normalised CoASH and CoA ester levels [24]. A further piece of theoretical evidence comes from computational models of the mFAO in rodents and humans: model simulations predicted that the loss of MCAD activity causes a severe decrease in CoASH, due to its sequestration into medium‐chain acyl‐CoAs [27, 28]. Simulations further suggest that the mFAO pathway is particularly vulnerable to the CASTOR phenotype in the case of substrate overload and/or specific enzyme deficiencies [29]. This is due to a vicious cycle, in which a little bit of acyl‐CoA accumulation exerts a product inhibition on the mFAO, thereby exacerbating acyl‐CoA accumulation and CoA sequestration. [29].

Quantitative and simultaneous analysis of acyl‐CoA esters and CoASH is challenging due to their structural complexity, such as the large variation in carbon‐chain length, degree of saturation, and the presence of functional groups. Moreover, CoA esters are highly susceptible to hydrolysis [30]. Recently, hydrophilic interaction liquid chromatography (HILIC) coupled with tandem mass spectrometry (HILIC‐MS/MS) enabled a comprehensive analysis of CoASH and short‐ to long‐chain acyl‐CoA (chain lengths ranging from 2 to 18 carbon atoms) in a single analytical run with good linearity, precision, and recovery [31]. This method allows us to explore the CASTOR hypothesis in MCADD experimentally in a convenient and direct way.

The sequestration of CoASH into CoA esters is not the only factor influencing CoASH levels. First, CoASH sequestration may be relieved by hydrolysis via thioesterases [32] or conversion into the cognate carnitine esters by carnitine acyltransferases [33, 34, 35]. Second, *de novo* biosynthesis of CoA from pantothenate (vitamin B5) is allosterically regulated by acyl‐CoA and acylcarnitine species, intermediates of the mFAO [36, 37, 38]. The first enzyme in the CoA *de novo* biosynthesis is pantothenate kinase (PANK). Humans have 3 genes encoding 4 active isoforms of PANK: PANK1α, PANK1β, PANK2 and PANK3. Their activity regulation differs, as well as their tissue‐specific expression levels (reviewed in [39, 40]). Particularly the PANK3 isoform, which is expressed in the liver, is inhibited by medium‐chain acyl‐CoA species, the metabolites that accumulate in MCADD. Third, local CoASH levels may be affected by the rerouting of acyl‐CoA into the peroxisomal ß‐oxidation [41, 42]. The mitochondrial transporters SLC25A16 [43] and SLC25A42 [44] and the peroxisomal transporter SLC25A17 (35) are thought to be involved in the transport of CoA between cellular compartments. Finally, the network of CoA release, biosynthesis, and redistribution (Fig. 1B) is regulated by gene expression in response to feeding/fasting cycles [45, 46], cold [47], and pharmacological intervention (e.g. HoPan) [34]. In summary, many factors of an interacting network influence the levels of CoASH and acyl‐CoAs in cells. It is currently unknown how this network responds to CASTOR diseases, such as MCADD.

Using *in silico*, *in vitro*, and *in vivo* models of MCAD deficiency and a new method to quantify CoASH and acyl‐CoAs in cells [31], we investigated if the loss of MCAD leads to the accumulation of medium‐chain acyl‐CoA esters, sequestration of CoASH, and adaptation of CoA metabolism. *In silico* and in *vitro*, we found that the loss of MCAD led to CoASH sequestration into C8‐acyl‐CoA, in agreement with the CASTOR hypothesis. While the quantification of CoASH in *in vivo* liver samples was not technically possible, we observed that, under severe energetic stress (fasting plus cold stress), MCAD‐KO mice upregulated their CoA biosynthesis and release pathways. Computational modelling showed that this adaptive response of CoA metabolism acts as a compensatory adaptation to relieve the CASTOR phenotype.

## Results

### MCAD deficiency leads to the sequestration of CoA into medium‐chain acyl‐CoA esters

**Fig. 2:**
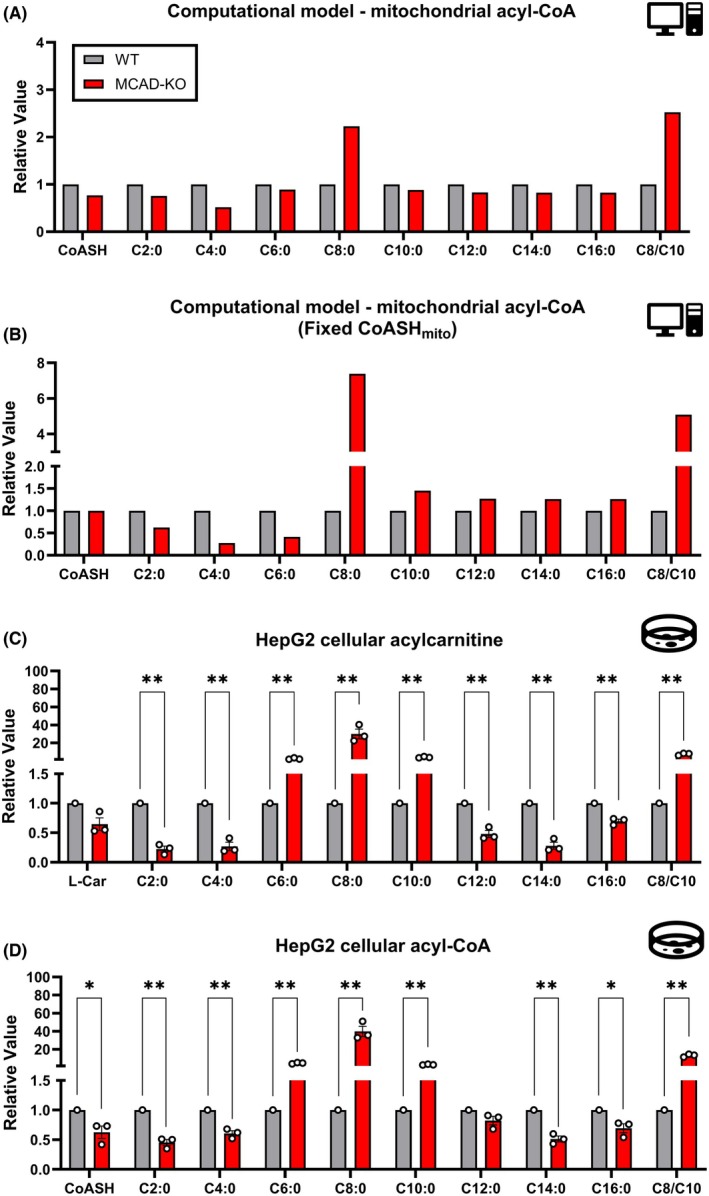
Acylcarnitine and ‐CoA profile in the computational model and HepG2 cells. (A) Mitochondrial acyl‐CoAs predicted by computational simulations of mitochondrial fatty acid oxidation (mFAO) in human liver (cf. [28]). (B). Mitochondrial acyl‐CoAs predicted by computational simulations with free mitochondrial coenzyme A (CoASH) fixed at 0.6 mm. (C) Acylcarnitine esters and D. Acyl‐CoA esters measured in HepG2 whole‐cell lysates. Wild‐type (WT) and MCAD‐knockout (MCAD‐KO) cells were cultured for 24 h in DMEM without glucose, pyruvate and glutamine, and supplemented with 0.5 mm palmitate and 2 mm L‐carnitine. Each data point represents the mean of 2–3 independent experiments of wild‐type (grey bar) or a different CRISPR/Cas9 MCAD‐KO clone (red bar). In turn, each independent experiment consisted of 4–5 parallel cell cultures/technical replicates. (Error) bars represent standard error of the mean (SEM); data are shown relative to WT; *P < 0.05 in 5 out of 9 experiments, **P < 0.01 in 5 out of 9 experiments. Expanded data set in Figs S2–S4. Statistics in Fig. S4, calculated using one‐way Brown‐Forsythe ANOVA adjusted by Dunnett's T3 multiple comparisons test. The groups (C2:0) indicated on the x‐axis refer to various lengths of the saturated acyl‐CoAs and ‐carnitines.

We first investigated how complete deficiency of MCAD activity would affect the concentrations of CoASH and acyl‐CoA esters. To this end, we adapted a previously constructed and experimentally validated computational model of the human liver mFAO, based on detailed kinetic properties of all enzymes [28] (Fig. 1A). Model simulations predicted a large increase in C8:0‐acyl‐CoA concentration in the MCAD‐KO relative to the wild‐type (WT) (Fig. 2A), while all other acyl‐CoA ester concentrations were unchanged or decreased (Fig. 2A). In agreement with the CASTOR hypothesis, CoASH was decreased in the MCAD‐KO relative to WT.

The model prediction that C8:0‐acyl‐CoA would be elevated, and C2:0‐ and C4:0‐acyl‐CoA (acetyl‐ and butyryl‐CoA) decreased in the MCAD‐KO was intuitive, as these compounds are the main substrate and downstream products of MCAD, respectively. It was surprising, however, that long‐chain acyl‐CoAs were decreased in the simulation: in a linear metabolic pathway, one would expect an accumulation of metabolites upstream of the deficient enzyme. The mFAO is not a simple linear pathway, though, as each intermediate metabolite sequesters a CoASH molecule, so consecutive reactions and cycles of mFAO compete for a potentially limiting substrate. Since the first enzymatic reaction of the mFAO in the mitochondrial matrix (CPT2) requires CoASH (Fig. 1A), the accumulation of medium‐chain acyl‐CoAs downstream could inhibit the CPT2, causing a decreased influx of long‐chain acyl‐CoAs: this leads to the counterintuitive scenario that a downstream bottleneck limits the rates of upstream reactions.

To test whether CoASH depletion was indeed causing the decrease in long‐chain acyl‐CoAs, we artificially fixed CoASH in the computational model, effectively giving CPT2 an unlimited CoASH supply (Fig. 2B). In this hypothetical situation, the loss of MCAD indeed led to the accumulation of long‐chain acyl‐CoAs, as one would expect upstream of a bottleneck (Fig. 2B).

These predictions were tested *in vitro* using an existing CRISPR/Cas9‐derived MCAD‐KO HepG2 cell line [28]. The absence of MCAD in the knockouts was confirmed by Western blot (Fig. S1). The WT and the three MCAD‐KO clones were then incubated in a medium without glucose, pyruvate and glutamine, in the presence of mFAO substrates palmitate and L‐carnitine. All MCAD‐KO clones showed elevated C6:0‐, C8:0‐ and C10:0‐acylcarnitine levels and a 6‐ to 8‐fold increased C8/C10 acylcarnitine ratio relative to the WT (Fig. 2C), in agreement with the diagnostic phenotype of MCADD patients [48]. Each data point in Fig. 2C,D represents a different MCAD‐KO clone, with at least 3 biological replicates (independent experiments), in turn consisting of 3–5 technical replicates (parallel cultures) (Figs S2–S4). The increase of C8:0‐acylcarnitine in MCAD‐KO versus WT cells was significant in all experiments (Fig. S4). Other medium‐chain acyl‐CoA concentrations (C6:0, C10:0) were increased significantly in most biological replicates (Fig. S4). The decrease of short‐chain acylcarnitines and saturated long chains up to C14:0 was also clearly significant.

To link these observations to the MCAD‐KO, the effect of overexpressing the *ACADM* gene in the knockouts was investigated. Western blotting confirmed the reintroduction of the MCAD protein (Fig. S1). Then it was confirmed that the C8:0‐acylcarnitine level was increased in the MCAD‐KO cells to which MCAD had been reintroduced, demonstrating that the phenotype was caused by the MCAD deficiency (Fig. S1).

The acyl‐CoA levels in the MCAD‐KO versus WT cells showed a similar pattern to that of the acylcarnitines (Fig. 2D). C6:0‐ and C8:0‐acyl‐CoA were significantly and consistently increased, C8‐acyl‐CoA by as much as 30 to 50‐fold (Fig. 2D). Interestingly, and as predicted by the computational model, C2:0‐, C4:0 and C14:0‐acyl‐CoA were decreased in most experiments. While C12:0‐acylcarnitine was significantly decreased, C12:0‐acyl‐CoA was not. CoASH, the molecule of interest in this study, was significantly lower in 5 out of 9 biological replicates (Figs S3 and S4).

C8:1‐acylcarnitine, a relevant biochemical marker of MCADD, and its cognate acyl‐CoA ester were mostly under the limit of detection and therefore excluded from the analyses. To further substantiate the results, the experiment was replicated in a culture medium in which not only mFAO substrates but also glucose, pyruvate, and glutamine were present, with essentially the same results (Figs S5–S7).

We concluded that the loss of MCAD activity leads to an increase of medium‐chain acyl‐CoA esters, and a decrease of CoASH, as well as short‐ and long‐chain acyl‐CoA levels. This supports the predicted sequestration of CoA into medium‐chain acyl‐CoA esters, to the extent that CoASH levels decrease and thereby most likely become limiting.

### Loss of MCAD does not affect CoA biosynthesis in cell culture

**Fig. 3:**
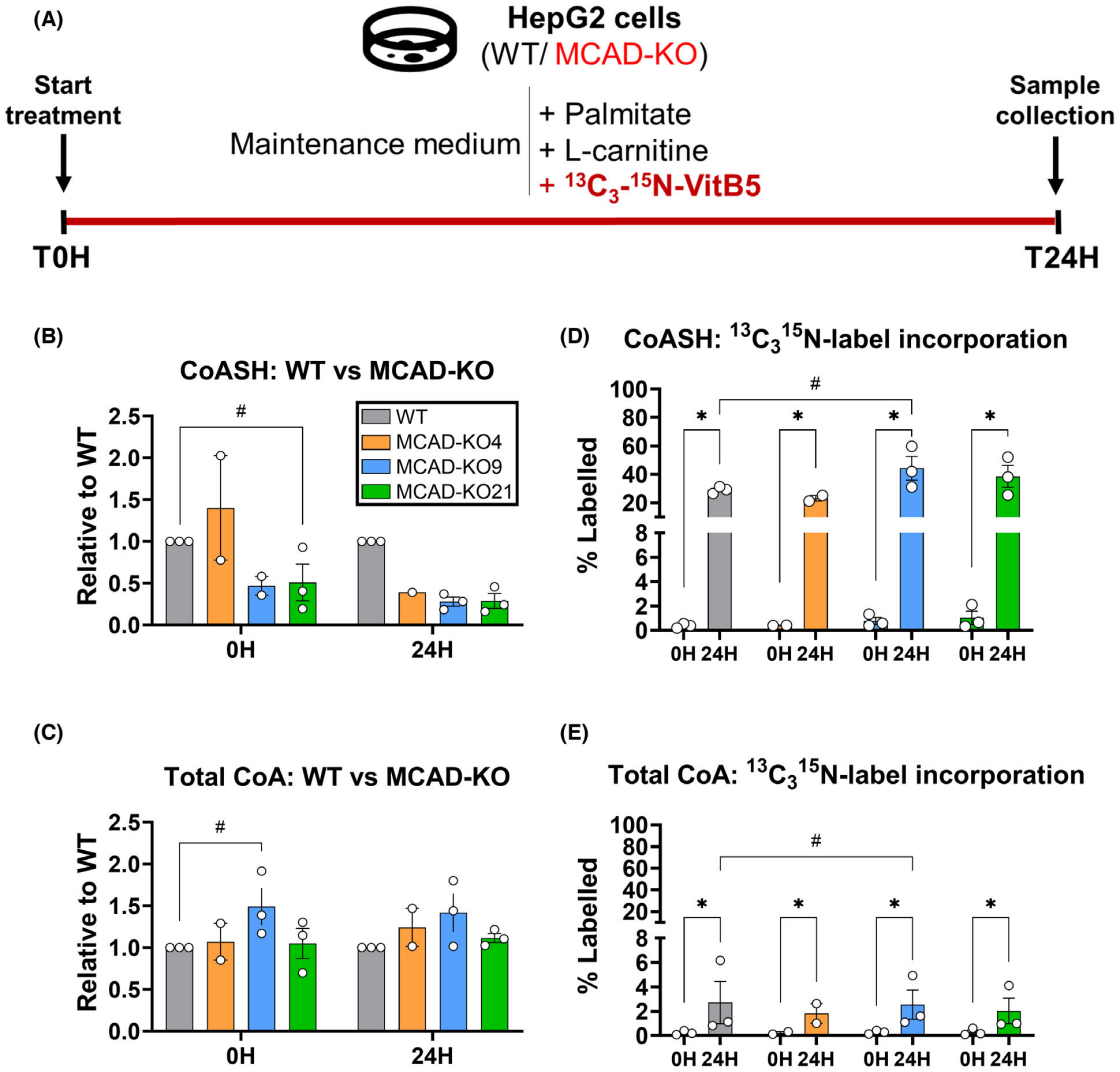
CoA biosynthesis in MCAD‐KO. (A) Simplified scheme of study design. Cells were treated with maintenance medium supplemented with 0.5 mm palmitate and 2.0 mm L‐carnitine, in which non‐labelled pantothenate was replaced by 4 mg·L^−1^ stable‐isotope‐labelled pantothenate (^13^C_3_‐^15^N‐VitB5). After 24 h samples were collected. (B, C) Free and total coenzyme A (CoASH and Total CoA, respectively) in MCAD‐knockout (MCAD‐KO) cells relative to wild‐types (WT) in the same experiment (*n* = 1–3; 3 independent experiments performed, data from failed experiments discarded). Statistics calculated using one‐way Brown‐Forsythe ANOVA adjusted by Dunnett's T3 multiple comparisons. (D, E) Percentage label incorporation into the free and total CoA pools. * when *P* < 0.05 versus T0H in 2 out of 3 experiments; # when *P* < 0.05 versus WT in 2 out of 3 experiments. Statistics calculated using atwo‐way ANOVA adjusted by Sídák's multiple comparisons test. For all experimental data (B–E), each data point represents an independent experiment, using a different cell passage (*n* = 1–3). In turn, each individual experiment consisted of 4–5 technical replicates that is simultaneous cell cultures; Error bars represent ± standard error of the mean (SEM). Statistics in Tables S2–S4.

It has been reported that PANK3, an important and highly expressed liver isoform of the first enzyme in the CoA biosynthetic pathway, can be inhibited by CoASH, C2‐acyl‐CoA and medium‐chain acyl‐CoAs [36, 37, 38]. Medium‐chain acyl‐CoAs were elevated (Fig. 2C), while C2‐acyl‐CoA and CoASH were decreased in the MCAD‐KO cells (Fig. 2D; Figs S3 and S4), providing a simultaneous inhibitory and stimulating effect. Given these potentially opposing mechanisms, we investigated whether CoA biosynthesis was affected by the knockout of MCAD in HepG2 cells. At the start of the experiment (T0H), the mFAO substrates palmitate and l‐carnitine were added and pantothenate, the precursor of CoA, was replaced by isotopically labelled ^13^C_3_^15^N‐pantothenate (Fig. 3A).

In agreement with Fig. 2D, an average decrease in CoASH in the MCAD‐KO relative to the WT cells was observed of approximately 3‐fold, albeit not significantly (Fig. 3B). WT and MCAD‐KO cells showed similar trends towards increased total CoA pool size (calculated as the acylated plus free fraction) and decreased CoASH at 24H relative to T0H levels (Fig. S8A,B, Table S1). However, the total CoA pool did not differ consistently between WT and MCAD‐KO cells either before or after incubation with mFAO substrates (Fig. 3C, Table S2). Similarly, the label incorporation in the total CoA pool was consistently very low, at an average of only 1–3% over 24 h for both WT and MCAD‐KO clones (Fig. 3E). Together this indicates that the MCAD‐KO cells do not have either up‐ or downregulated CoA biosynthesis compared to WT cells.

Remarkably, in all MCAD‐KO and WT cells, the relative label incorporation was approximately 10 times higher in the CoASH fraction than in the total CoA pool (Fig. 3D,E), suggesting the existence of a large inert CoA pool that is replaced at a much slower rate than the free CoASH fraction.

In conclusion, the fact that label incorporation and total CoA were not consistently affected by the MCAD‐knockout clones suggests that loss of MCAD did not significantly affect the CoA biosynthesis rate in these HepG2 cells under the conditions studied.

### MCAD‐KO mice remodel CoA metabolism under severe energetic stress

**Fig. 4:**
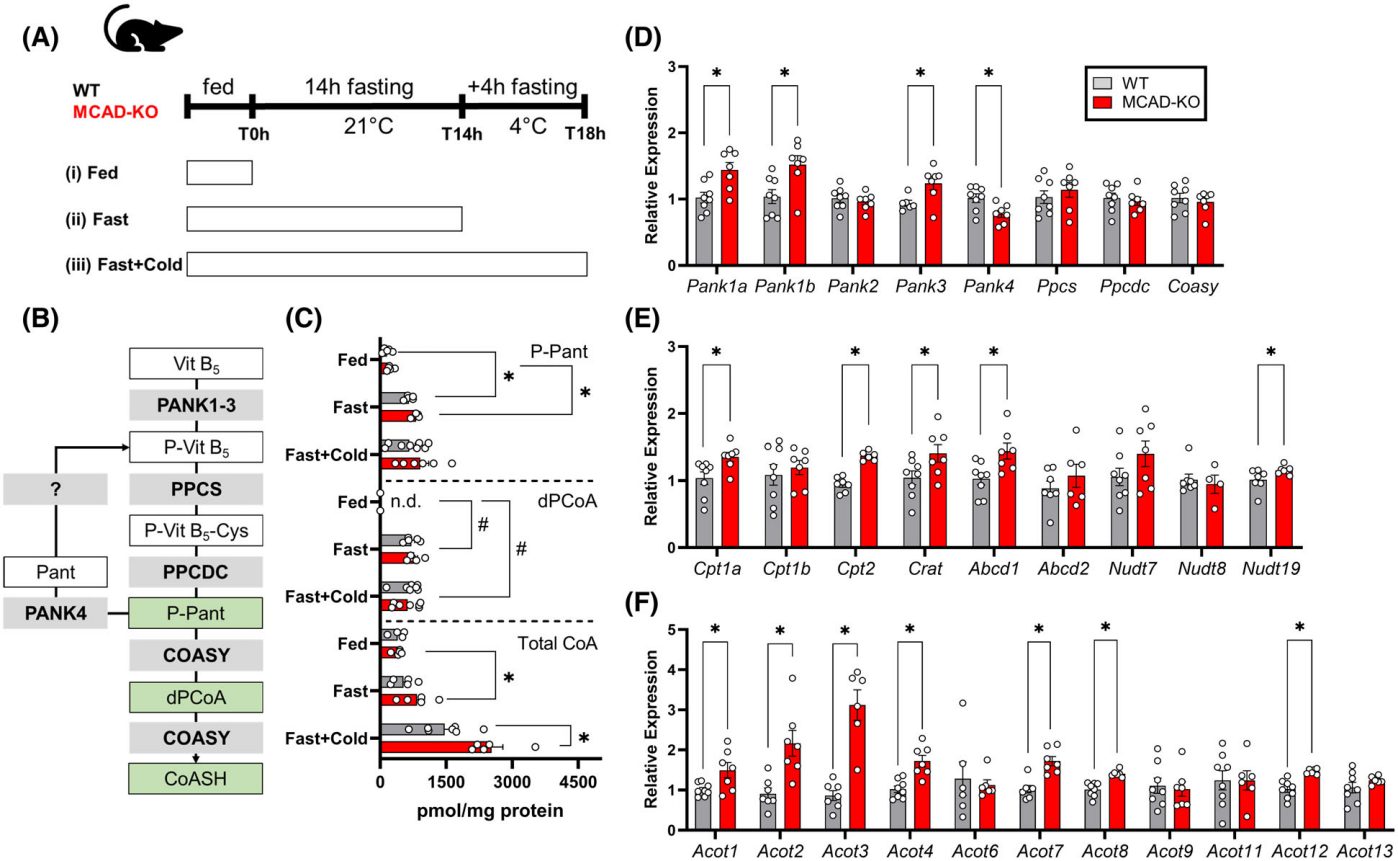
CoA metabolism in the liver of mice exposed to a combination of fasting and cold. (A) Mice had free access to food (fed), were fasted overnight for 14 h (fasted), or fasted for 18 h of which the last 4 h at 4 °C (fasted + cold). (B) Coenzyme A (CoA) biosynthesis pathway. PANK (gene *Pank*), pantothenate kinase; PPCS (gene *Ppcs*), phosphopantothenate‐cysteine ligase; PPCDC (gene *Ppcdc*), phosphopantothenoylcysteine decarboxylase; COASY (gene *Coasy*), bifunctional coenzyme A synthase; Vit B_5_, pantothenate; P‐Vit B_5_‐, phosphopantothenate; P‐Vit B_5_‐Cys, phosphopantothenoylcysteine; P‐pant, 4‐phosphopantetheine; Pant, pantetheine; dPCoA, dephospho‐CoA. Metabolites are shown in white or green boxes (green boxes correspond to data in panel C) and enzymes in grey. (C) Metabolite concentrations in the CoA biosynthesis pathway. (D–F) Hepatic mRNA levels encoding CoA biosynthetic enzymes (D), acyltransferases and peroxisomal enzymes (E), and acyl‐CoA thioesterases (F) in fasted, cold‐exposed MCAD‐knockout (MCAD‐KO, *n* = 8) mice relative to average wild‐type (WT, *n* = 8) levels (cf. Fig. 1B). *P < 0.05 using to an unpaired t‐test; # below limit of detection in fed mice, not in the other conditions; n.d., not detected; ± standard error of the mean (SEM). Genes abbreviated as follows (Note: for some there are multiple isoforms): *Cpt*, carnitine palmitoyltransferase; *Crat*, carnitine acetyltransferase; *Abcd*, ATP‐binding cassette domain transporter; *Nudt*, nudix hydrolase (nucleoside diphosphate linked moiety X‐type motif); *Acot*, acyl‐CoA thioesterase.

In MCADD patients, metabolic decompensations are typically triggered by energetic stress, such as fasting combined with cold exposure or febrile illness. We therefore wondered how energetic stress would affect CoA metabolism in a mouse model. In a previous study, MCAD‐KO mice exhibited elevated organic acid concentrations and a reduced fasting plasma glucose concentration [49]. When exposed to a combination of fasting and cold (4 °C), young MCAD‐KO mice could not maintain their body temperature and showed a higher death rate than WT mice [49]. We therefore set out to investigate hepatic CoA metabolism in young (8‐week‐old) MCAD‐KO mice upon fasting and cold exposure. WT and MCAD‐KO littermate mice were split into three groups: (i) fed, (ii) 14 h‐overnight‐fasted, or (iii) 14 h‐overnight‐fasted at room temperature plus a further 4 h of fasting at 4 °C (making a total of 18 h fasting) (Fig. 4A). Both MCAD‐KO and WT mice showed a significant decrease in body temperature after fasting + cold exposure (Fig. S9A). Blood glucose, however, was significantly reduced in MCAD‐KO *versus* WT mice after fasting + cold exposure, pointing to increased vulnerability of the mutant under energetic stress (Fig. S9B).

Hepatic levels of 4‐phosphopantetheine (P‐Pant), an intermediate in the CoA biosynthesis pathway, were increased in fasted compared to fed mice, in both WT and MCAD‐KO mice (Fig. 4B,C). A similar pattern was observed in the total CoA level, albeit only significant in the MCAD‐KO group. Dephospho‐CoA (dpCoA) was only measurable in fasted and fasted and cold‐exposed mice; it was below the limit of detection in fed mice, hence also elevated by fasting (Fig. 4B,C). Most importantly, in fasted and cold‐exposed MCAD‐KO mice, total CoA levels were on average 73% higher than in the corresponding WT group (Fig. 4C).

Unfortunately, it was not possible to quantify distinct concentrations of the different acyl‐CoA esters or the CoASH in these mice. When applying the same HILIC‐MS/MS method [31] as used above to mouse liver samples, we encountered technical challenges, such as low recovery and high variability in the data.

Subsequently, we investigated if the elevated total CoA levels in MCAD‐KO mice relative to WT under energetic stress were due to altered biosynthesis. Indeed, average mRNA levels encoding isoforms and paralogs of PANK (*Pank1a, Pank1b* and *Pank3*), the first enzyme in the CoA biosynthesis pathway, were upregulated by 24–53% in MCAD‐KO relative to WT mice when exposed to fasting plus cold (Fig. 4D). The mRNA encoding *Pank4*, an enzyme that counteracts CoA biosynthesis by converting 4‐phosphopantetheine back to pantetheine [50], was significantly downregulated (Fig. 4D). At the same time, the mRNA levels encoding the carnitine acyltransferases *Cpt1a*, *Cpt2* and *Crat* were upregulated, on average, by 35–41% in MCAD‐KO relative to WT mice (Fig. 4E). These enzymes can release mitochondrial CoASH by transferring acyl‐groups to L‐carnitine, forming acylcarnitines, which can cross the mitochondrial membrane (Fig. 1A) [51, 52]. A potential downstream effect of this can be seen in the increased medium‐chain blood acylcarnitines in MCAD‐KO mice, while the medium‐chain acylcarnitines in the liver are similar between WT and MCAD‐KO subjected to fasting and cold exposure (Fig. S9C,D). This is consistent with a previous study that showed increased circulatory acylcarnitines in mice exposed to cold stress [53].

The mRNA encoding the peroxisomal acyl‐CoA transporter *Abcd1* was also upregulated. Entry of acyl‐CoA into the peroxisomes may bypass the deficient MCAD enzyme, thereby also alleviating the burden on the mitochondrial CoA pool [41, 54]. We note that the transport mechanism of ACBD1 is still under discussion, but that all proposed mechanisms would relieve the mitochondrial CoA sequestration [55, 56]. Upregulation of *Nudt19*, potentially involved in detoxification of acyl‐CoA esters (Fig. 1B), was statistically significant (Fig. 4E). Finally, mRNA levels of several acyl‐CoA thioesterase (ACOT) isoenzymes were substantially and significantly upregulated (Fig. 4F), with even a 2‐ to 4‐fold increase of *Acot2* and *Acot3* expression. The corresponding ACOT2 protein is located in the mitochondrial matrix, where an accumulation of mFAO intermediates is likely to have a large effect; that said, it is present only at relatively low levels in the liver [32, 57]. Smaller, but still significant changes were seen for mRNAs encoding other ACOT isoenzymes with a median increase between 40% (*Acot8*) and 64% (*Acot*4 and *Acot7*). ACOT7 is especially relevant to our research question, as it constitutes a large part of thioesterase activity in the human liver and is also mitochondrial [32, 57]. Collectively, thioesterases offer a mechanism for rescuing CoASH levels, as they cleave acyl‐CoA esters to yield free fatty acids and CoASH.

In the HepG2 cells, Pank3 mRNA was elevated in the ‘palmitate, no glucose’ medium compared to ‘palmitate, low glucose’, which was significant in WT and MCAD‐KO‐clone 4 (Fig. S10). This suggests that the additional stressor of removing glucose altogether does have an effect on CoA metabolism, although the difference was not seen in the acyl‐CoA pattern nor in the free CoASH pool (Fig. S7). Similarly, no clear pattern of altered gene expression could be observed between MCAD‐KO and WT cells (Figs S11–S14), in line with the identical total CoA and CoASH labelling pattern in mutant and WT cells. It is worth noting that MCAD‐KO9, the clone which displayed an increased percentage label incorporation into the CoA pools (Fig. 3D,E), presented a consistent upregulation of *Acot8* and *Acot13* (Figs S11–S14).

Altogether, the changes in gene expression observed in the liver of MCAD‐KO mice exposed to fasting and cold work towards increasing the CoA synthesis, releasing CoASH, and relieving the accumulation of acyl‐CoA species (most strikingly via the ACOTs). This effect was specifically observed under severe energetic stress.

### Combined upregulation of CoA synthesis and ACOT normalise CoA metabolites

**Fig. 5:**
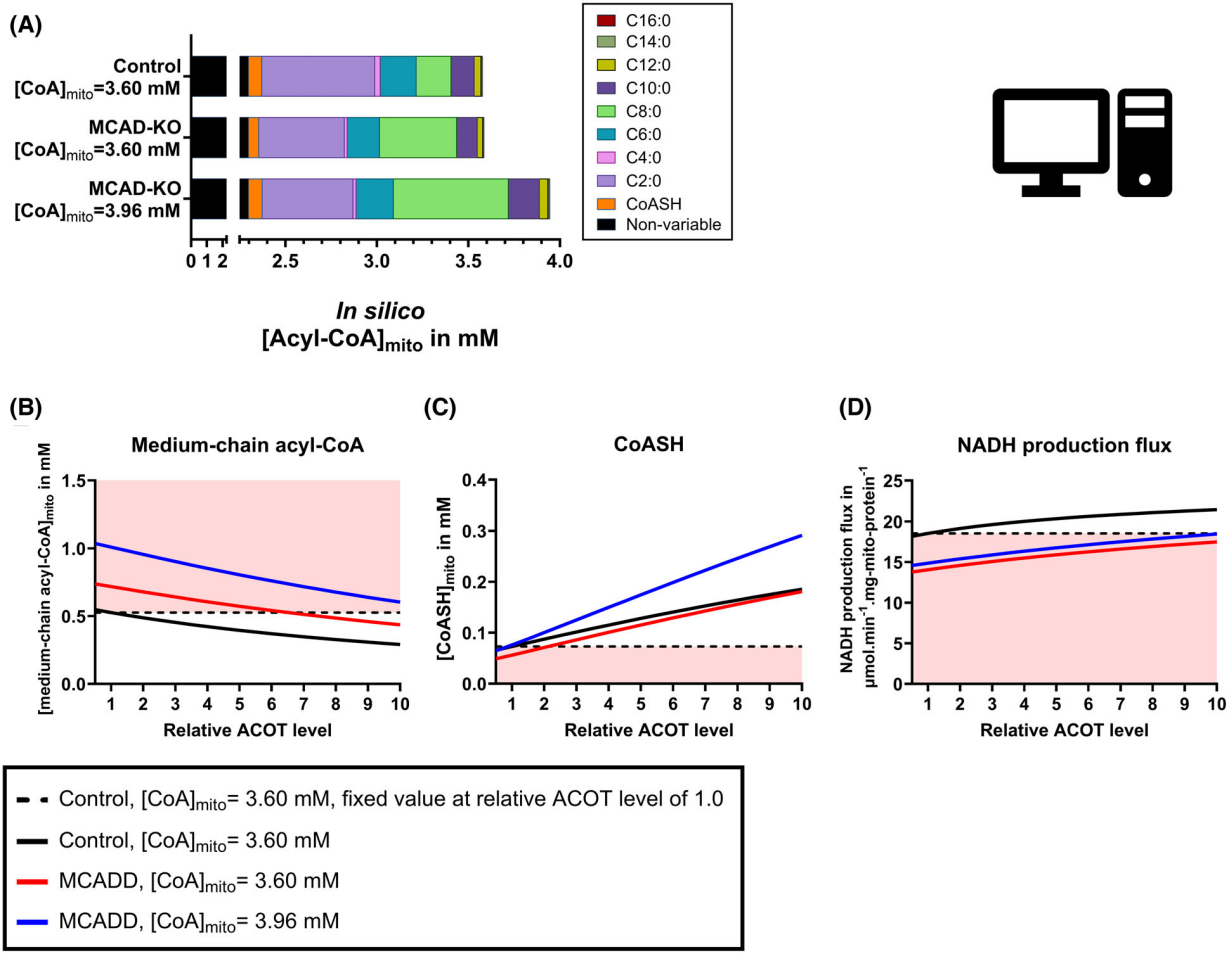
*In silico* analysis: increased total CoA and ACOT. Three models are compared: Control (3.6 mm coenzyme A, abbreviated CoA), MCAD‐knockout (‐KO) (0% MCAD activity, 3.6 mm CoA), and MCAD‐KO with elevated total CoA (3.96 mm). (A) Distribution of acyl‐CoA esters (the legend indicating individual chain lengths) over the mitochondrial CoA pool. The pool considered non‐variable in the model (black) represents a realistic amount of CoA that would be sequestered by mitochondrial pathways other than the mitochondrial fatty acid oxidation, including as succinyl‐CoA and propionyl‐CoA. (B, C) Steady‐state mitochondrial concentrations of (B) medium‐chain acyl‐CoAs and (C) free CoA (CoASH) at increasing acyl‐CoA thioesterase (ACOT) activity, relative to the default ACOT *V*_max_ of 0.003 μmol·min^−1^·mg‐mito‐prot^−1^; (D) NADH production flux. As a reference for the control condition, the control value at relative ACOT level of 1.0 was fixed and displayed in the graphs B–D as a dashed line; the pink filled area represents the range outside the reference control condition.

In order to understand at a metabolic level what the measured increase in *Acot* expression and total CoA pool size would mean in MCADD, we turned again to the computational model. To mimic energetic stress in the kinetic model, we used a high concentration of palmitate, simulating lipid mobilisation during, for instance, fasting or cold exposure [58]. As shown before (Fig. 2), in the MCAD‐KO computational model, C8‐acyl‐CoA sequestered a large fraction of the available CoA, resulting in a reduced CoASH fraction. Interestingly, if the total CoA in the MCAD‐KO was increased in the computational model by 10% (Fig. 5A), the CoASH was completely restored to the WT level. Most of the extra CoA, however, ended up in the C8‐acyl‐CoA fraction, which may be toxic [59].

To simulate the effect of increased *ACOT* expression, the maximal catalytic capacity (*V*_max_) of ACOT was systematically varied. Again, at low ACOT activities (0.5–1 relative to the reference value), when total CoA was increased by 10%, the levels of medium‐chain acyl‐CoAs in the MCADD computational model increased by 40% (Fig. 5B). Concomitantly, the CoASH fraction also increased to a similar extent in MCADD (by 36%), restoring its concentration to control levels (Fig. 5C). Increased ACOT activity effectively lowered medium‐chain acyl‐CoAs, while at the same time increasing CoASH. The combined effect of elevated total CoA and ACOT activity was more effective than either alone in enhancing the mFAO flux in MCADD, albeit only by 5% (Fig. 5D). A 10‐fold increase in ACOT activity completely normalised the mFAO flux and brought the medium‐chain acyl‐CoAs down to control levels. Together, these results show how the combined upregulation of total CoA and *ACOT* observed in mice (Fig. 4) can efficiently relieve the CASTOR phenotype in MCADD and normalise CoASH and medium‐chain acyl‐CoA levels to those observed in controls.

## Discussion

This study provides, for the first time, direct evidence that an MCAD deficiency can lead to a decrease in CoASH, which coincides with a large accumulation of C8‐acyl‐CoA. Experimental evidence for the predicted CoASH depletion in MCADD was lacking so far. In a mouse model of propionic acidaemia, a reduced hepatic CoASH and concomitant accumulation of propionyl‐CoA had been demonstrated before [24, 25]. Thus, to our knowledge, MCADD is the second disease, after propionic acidaemia [25], for which the CASTOR mechanism has been validated experimentally. We showed this in a variety of disease models, *in vitro*, *in vivo*, and *in silico*. Moreover, this is to our knowledge the first report showing extensive compensatory adaptation of CoA biosynthesis and CoA release to a CASTOR disease. Interestingly, this adaptive response was most pronounced in MCAD‐KO mice under severe energetic stress, whereas CoA biosynthesis was unaltered in MCAD‐KO HepG2 cell cultures. Computational simulations showed that the combined increase of total CoA and ACOT activity that was observed in the MCAD‐KO mice can interact to provide effective protection against CoASH depletion and toxic C8:0‐acyl‐CoA accumulation, as well as restore pathway flux.

Since MCAD prefers C8:0‐acyl‐CoA as substrate [10, 60], the observed accumulation of C8‐acyl‐CoA and the tendency to a decline in CoASH are the most direct validation of the CASTOR hypothesis for MCADD. Interestingly, both long‐ and short‐chain acyl‐CoAs were decreased *in silico* and *in vitro*. In a linear pathway, one would expect longer‐chain acyl‐CoAs, upstream of the deficient MCAD enzyme, to accumulate. The most likely explanation might be that the entry of long‐chain acyl‐CoAs into the pathway is hampered by the reduced availability of CoASH, which is required as a co‐substrate in the carnitine shuttle [61]. This intuition was confirmed in a simulation with fixed free mitochondrial CoASH (Fig. 2B). If unfixed, the concentration of mitochondrial CoASH in the computational model declines to levels (225.1 μm for the WT *versus* 137.1 μm in the MCAD‐KO) well below the reported K_m_ of CPT2 for CoASH in human cells (*K*_*m*_ = 1300–1400 μm [62, 63]), suggesting that the altered CoASH concentration declines within a sensitive range for CPT2 activity. The implications for an MCADD patient might be dire: It would mean that when fatty acids are the main substrate, most of the acyl‐CoA esters are medium chains, a toxic metabolite [64] of which the oxidation is impaired, all the while hampering the importation of chain lengths for which the oxidation is more functional.

Also noteworthy is the similarity of the acylcarnitine and acyl‐CoA profiles (Fig. 2C,D). Increased plasma C8:0‐acylcarnitine in MCADD has been known for many decades [65] and is pivotal in diagnosis and newborn screening [11]. Different carnitine acyltransferase isoenzymes in various cellular compartments interconvert acyl‐CoAs and acylcarnitines [61]. It is therefore often assumed that acylcarnitine profiles in plasma reflect acyl‐CoA profiles in tissues [13, 66]; yet, this is rarely tested. This is not trivial, as differences in the patterns of acyl‐CoA and acylcarnitine chain lengths have been reported across tissues, compartments and even within compartments [67, 68, 69]. Against this background, it is reassuring to observe a clear correlation between high C8‐acylcarnitine and C8‐acyl‐CoA in MCADD cells.

Acetylcarnitine (C2:0‐acylcarnitine) constitutes the second‐largest fraction of the plasma carnitine pool (after free L‐carnitine), accounting for a third of total plasma carnitine in healthy, fasted children [70]. Both acetyl‐CoA (C2:0‐acyl‐CoA) and ‐acylcarnitine were decreased in MCAD‐KO versus WT HepG2 cells (Fig. 2). This is in contrast to earlier observations that MCADD patients had massively increased acetylcarnitine levels in urine during a metabolic decompensation (median: 75.6 μmol·mmol^−1^ creatinine; range: 8.11–98.28 μmol·mmol^−1^ creatinine), compared to 0.76 μmol·mmol^−1^ creatinine (range: 0.11–18.68 μmol·mmol^−1^ creatinine) under normal fed and 0.09 μmol·mmol^−1^ creatinine (range: 0.02–5.08 μmol·mmol^−1^ creatinine) under fasted conditions in the same patients [28]. In healthy children, the values range between 3.47–6.17 μmol·mmol^−1^ creatinine [71]. In the context of the CASTOR hypothesis, it is tempting to speculate that a limited availability of CoASH for the citric acid cycle hampers further oxidation of acetyl‐CoA into CO_2_ and leads to an overflow of acetylcarnitine into blood and urine. This is echoed by the ability of triheptanoin—a triglyceride containing only short‐chain fatty acids—to circumvent an MCAD deficiency and improve histological and biochemical indicators of liver pathology in MCAD‐KO mice [72]. To elucidate this, detailed multi‐compartment time‐course data under controlled conditions will be needed.

Various catabolic triggers, including fasting, high‐fat feeding and cold exposure, can increase the total CoA pool in mammalian liver [68, 73, 74]. Furthermore, cold exposure increased the rate of incorporation of labelled pantothenate in rat liver, suggesting an upregulated CoA biosynthesis [47]. This is consistent with our observation that the total CoA pool is increased in MCAD‐KO mice after fasting; moreover, the CoA pool is further increased upon fasting + cold exposure, both in WT and MCAD‐KO mice (Fig. 4). What is new from our work was the increased total CoA pool in fasted, cold‐exposed MCAD‐KO mice relative to WT littermates, in combination with upregulated expression of genes involved in CoA biosynthesis and release. We observed a significant upregulation in the gene expression of five processes that can increase the levels of CoASH and/or decrease the levels of potentially toxic C8‐acyl‐CoA in the mitochondrion: (i) CoA biosynthesis, (ii) ACOTs, (iii) carnitine acyltransferases, (iv) peroxisomal acyl‐CoA transporters and (v) NUDTs.

Tolwani *et al*. [49] exposed MCAD‐KO homozygotes to cold and a fasting challenge until death, and observed severe cold sensitivity, evidenced by a dramatic drop in body temperature in MCAD‐KO relative to WT mice. Given our interest in the trajectory of possible pathophysiology in MCADD patients, we terminated the experiment after 4 h of cold exposure. This might explain why we did not see a relative difference in body temperature between MCAD‐KO and WT mice (Fig. S9A). However, a clear and significant decrease in blood glucose of MCAD‐KO versus WT mice confirms that the MCAD‐KO mice were more energetically stressed than WT mice (Fig. S9B). The gene expression changes in MCAD‐KO relative to WT mice might, therefore, be interpreted as attempts at adapting the CoA metabolism to cope with a metabolic challenge. This can be seen as further support for the CASTOR hypothesis.

**Fig. 6:**
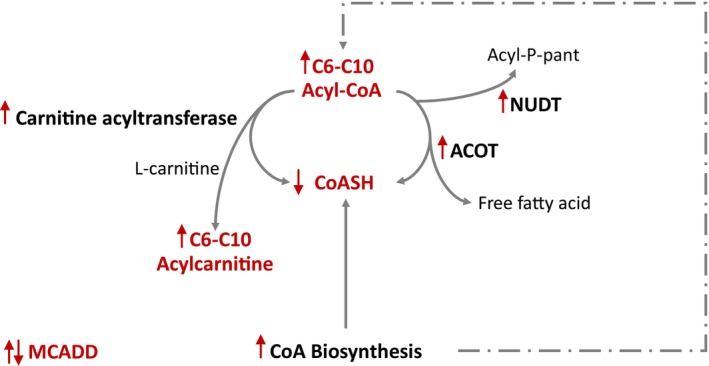
Hepatic changes in response to severe energetic stress in MCADD. The text and arrows in red indicate places where MCADD cells or animals behave differently from wild‐type (WT) cells under stress. Coenzyme A (CoA) biosynthesis can increase the free CoA (CoASH) pool by producing more CoA. A dashed line shows a possible side‐effect of this, in that medium‐chain acyl‐CoAs (approximately chain lengths C6–C10) would also further accumulate in response. Acyl‐CoA thioesterases (ACOTs) and carnitine acyltransferases free up existing CoASH from acyl‐CoAs, while at the same time preventing accumulation of acyl‐CoAs. Lastly, nudix hydrolases (NUDTs) destroy a CoA by converting an acyl‐CoA to an acyl‐4‐phosphopantetheine. This might reduce the levels of toxic acyl‐CoA intermediates. Several enzymes that participate in these processes were upregulated in MCAD‐KO mouse liver, which might constitute an attempt at increasing CoASH and decreasing medium‐chain acyl‐CoAs.

Fig. 6 summarises the possible anti‐CASTOR effects of the adaptive response of CoA metabolism that was observed in the *in vivo* model under energetic stress. The largest, most compelling adaptation was the increased expression of seven different *Acot* genes, of which at least two (*Acot2* and *Acot7*) are abundant in the liver mitochondria [32, 57]. Another consistent trend was the upregulation of *Pank* expression. PANKs catalyse the first reaction in the CoA biosynthesis [75, 76] and their upregulation is consistent with the observation that the total CoA pool was elevated. Since the combination of upregulated total CoA and ACOT activity would alleviate the decline of CoASH and the accumulation of potentially toxic C8‐acyl‐CoA, while simultaneously restoring pathway flux to control levels (Fig. 5), it could be a protective mechanism in patients. While the difference in pathway flux between the models with and without an increase in total CoA is small (5%), the increased availability of CoASH would also stimulate other energy‐yielding pathways, such as the branched‐chain amino acid oxidation [77]. Moreover, the K_m_ values of many CoASH‐consuming mitochondrial enzymes are within the predicted range of CoASH concentrations (50–200 μm) for example branched‐chain α‐keto acid dehydrogenase [78, 79], pyruvate dehydrogenase [79, 80], and α‐ketoglutarate dehydrogenase [80]. This suggests that they would be stimulated by substrate increases in this range.

It remains to be investigated if patient‐specific differences in corrective responses of CoA metabolism underlie the differences between symptomatic and asymptomatic individuals who are homozygous for the same c.985A>G missense mutation in the *ACADM* gene. It would also be interesting to explore the regulation of CoA metabolism in other (putative) CASTOR diseases. Depending on the substrate specificity of the thioesterases involved [32], a similar response could, in principle, provide protection in each of them.

The incorporation of labelled pantothenate (vitamin B5) into CoA revealed a conspicuous discrepancy between the turnover of non‐esterified CoASH versus total CoA (Fig. 3F,G). After 24 h, around 20% of the CoASH pool was labelled, but less than 2% of the total CoA pool. The thioesterification and hydrolysis of CoA was previously reported to have a turnover time of less than a minute [8]. This implies that the free and acylated CoA pools should be in constant exchange, suggesting that the fraction of label in free and acylated CoA should be equivalent. Interestingly, it has been observed elsewhere, using the same labelled VitB5 as in the present study, that less than 5% of the CoA pool is labelled over 3 h in a breast cancer cell line, despite the full replacement of the intracellular VitB5 pool with its labelled counterpart [50]. It is worth noting that some fraction of the cellular CoA pool is also sequestered by binding to cysteine moieties in proteins (CoAlation) or by sulphide bridge formation with glutathione [81]. It is unclear whether these CoAs are as labile as the rest of the CoA pool.

We initially wondered whether CoA biosynthesis might be allosterically inhibited by the medium‐chain acyl‐CoAs that accumulate in the absence of MCAD. In particular, PANK3 is inhibited by C8:0‐acyl‐CoA *in vitro* [36]. However, despite C8:0‐acyl‐CoA levels clearly being upregulated, we found no effect of MCAD‐KO on CoA biosynthesis in HepG2 cells. The intracellular situation is complex, since PANK paralogs and isoforms have different subcellular locations and different sensitivities to allosteric inhibitors [38, 82, 83]. For example, PANK3 and its mitochondrial paralog, PANK2, are both strongly inhibited by acetyl‐CoA (C2:0‐acyl‐CoA) [38, 82]. The reduced acetyl‐CoA in the MCAD‐KO cells may therefore stimulate PANK activity and counteract any inhibition by C8:0‐acyl‐CoA. Whatever the underlying mechanisms may be, our data do not suggest an important allosteric effect of C8:0‐acyl‐CoA on CoA biosynthesis in MCAD‐KO HepG2 cells under the conditions studied, despite a 30‐ to 50‐fold increase in C8‐acyl‐CoA.

A limitation of the current study is that the various disease models do not reflect all aspects of MCADD in patients. Kinetic models contain only a subset of the metabolic reactions, thus precluding the study of other compensatory or aggravating mechanisms. HepG2 cells, but also more advanced patient‐derived liver organoids [84, 85, 86, 87, 88, 89], barely produce glucose, which is an important aspect to understand MCADD symptoms. The MCAD‐KO mice give more insight into the physiology of the liver in the context of the whole body and into the response to energetic stress. An important limitation in characterising the underlying dynamics of fatty acid metabolism *in vivo* is the difficulty of measuring acyl‐CoAs in tissue samples. The current acyl‐CoA method [31], when applied to the mouse liver samples, exhibited significant technical challenges. We observed degradation in the internal standards, poor recovery, and significant variation in the samples. This could be attributed to the complexity of the tissue samples as compared to the HepG2 cells. The difficulties in analysing acyl‐CoAs in tissues have been reported by other studies as well [30, 90, 91] and dedicated sample preparation and method development were required for the quantification of acyl‐CoAs in the tissues. Addressing these challenges associated with tissue samples is beyond the scope of the current study and should be investigated in future research. Additionally, although better resembling the complexity of an MCADD patient than an *in vitro* cell line, mice do differ in important ways from humans, for instance having an additional long‐chain acyl‐CoA dehydrogenase (LCAD), which may partially compensate for the loss of MCAD [92]. Patient studies like FiTtINg MCADD (NCT03761693) will partially fill this gap by providing time‐course data in patients under controlled conditions [93], although they will only allow sampling of accessible body fluids. M Thus, a combination of patient and model data will remain necessary to obtain the full picture.

In conclusion, a combined *in silico*, *in vitro*, and *in vivo* approach demonstrated that the loss of MCAD triggers a CASTOR phenotype. Particularly under severe energetic stress, a number of rescue mechanisms were activated that could alleviate the decline of CoASH and accumulation of acyl‐CoA esters. This study thus paves the way for further investigations to study the regulation of CoA metabolism by complex mixtures of CoA esters *in vivo*, and to evaluate the impact of CoA availability on the pathogenesis of MCADD and other metabolic diseases.

## Materials and methods

### Computational modelling

Simulations were performed in Wolfram Mathematica version 13.1. A kinetic model of the mFAO in human liver [28] was extended with an acetyl‐CoA (C2:0‐acyl‐CoA) sink, allowing the prediction of mitochondrial acetyl‐CoA levels under various conditions. Simple first‐order kinetics were used:

$$v_{C2\text{AcylCoAsink}}=k_{C2\text{AcylCoAsink}}\cdot {\left[C2\text{AcylCoA}\right]}_{\text{mito}}$$

The sink constant (k_C2AcylCoAsink_ = 0.03 min^−1^ g‐mitochondrial‐protein^−1^) was fitted so the control model predicts a steady‐state acetyl‐CoA concentration of 600–800 μm, in accordance with measured liver acetyl‐CoA levels [94]. The concentration of the cytosolic substrate palmitoyl‐CoA was fixed at 150 μm, to mimic the concentration of cytosolic, long‐chain acyl‐CoAs previously reported [95]. The control values of total mitochondrial CoA (3600 μm) and the *V*_max_ of acyl‐CoA thioesterase (0.003 nmol min^−1^ mg protein^−1^) were the same as in the original model [28] and were perturbed as mentioned in Fig. 5. Models are available for simulation and download from the JWS Online model repository [96, 97] via the following link: https://jjj.bio.vu.nl/models/odendaal4/simulate/.

### Statistical analysis and data representation

Analysis of differences between two groups was done using an unpaired Student t‐test. For the comparison of three or four groups, we performed one‐way Brown‐Forsythe ANOVA followed by the Dunnett's T3 *post hoc* test. Briefly, Brown‐Forsythe ANOVA is a variation of the Ordinary ANOVA, which does not assume the same standard deviations across the groups. And the Dunnett's T3 test corrects for the multiple comparisons performed against one control group. When the samples passed the Brown‐Forsythe test, presenting the same standard deviation across groups, we used the two‐way ANOVA test adjusted by the Sídák's test for the multiple comparisons. These analyses were performed using GraphPad Prism version 9.1 for Windows (graphpad Software). The results were considered statistically significant when the *P*‐value was smaller than 0.05. All the mouse data (Fig. 4) are shown as mean ± standard error of the mean (SEM). Regarding the HepG2 cells (WT, MCAD‐KO4, MCAD‐KO9, MCAD‐KO21), the results were presented in a distinct way (Figs 2 and 3, S2–S8, S10, S1–S14). For all the HepG2 analyses, (I) the experiments were repeated up to three times, (II) individual experiments consisted of 4–5 technical replicates (cell cultures), and (III) the results obtained consisted of values relative to the WT group within each experiment, instead of absolute values. In other words, assuming the WT group to be 1, the values indicated how much the MCAD‐KO cells varied in relation to the WT. Consequently, the average of the WT group across experiments was 1, which did not allow for the performance of the statistical test of combined experiments due to the lack of variance within the WT. To circumvent this issue, we performed statistical analysis per experiment instead (*n* = 3). The significance scores and graphical visualisation of all the individual analyses, per experiment and per MCAD‐KO clone, are detailed in the supplement (Figs S4, S6, S12 and S14, Tables S1–S4).

### Cell lines

The HepG2 MCAD‐KO clones (KO4, KO9, KO21) were previously generated from a commercially available HepG2 cell line (RRID:CVCL_0027; order number: ATCC‐HB‐8065, ATCC, Manasses, Virginia, USA) [28].

### MCAD overexpression in MCAD‐KO HepG2 cells

HepG2 cells overexpressing the *ACADM* gene were generated using a lentivirus system, in which HEK293T cells (RRID:CVCL_1926; order number: ATCC‐CRL‐11268‐293T/22, ATCC, Manasses, Virginia, USA) were used as a lentivirus‐producer cell line.

#### Cell culture

HEK293T and HepG2 cells were cultured in DMEM with GlutaMAX (Gibco, 31 966–047, Waltham, MA, USA), 10% FBS (Hyclone, SV30160, Logan, UT, USA), and 1% Penicillin–Streptomycin (10 000 U·mL^−1^; Gibco, 15 140 122,), and kept at 37 °C, 5% CO_2_. Routine *Mycoplasma testing* was performed to ensure that all experiments are carried out with *Mycoplasma*‐free cells.

#### Virus transduction

A third‐generation lentiviral vector pGenLenti, expressing the human *ACADM* gene, was ordered from Genscript (U4143IC010). For empty vector control virus, the plasmid pCDH‐CMV‐MCS‐EF1 (System Biosciences, CD510B‐1, Palo Alto, CA, USA) was used. Lentivirus was produced in HEK293T cells. Virus‐containing medium was added to HepG2 cells at 24 h and 48 h after transfection. The lentiviral expression vector included puromycin resistance as a selection marker to select stably transduced polyclonal cells. Puromycin was used at a final concentration of 1.5 μg·mL^−1^.

### Protein extraction and immunoblotting

Cells were lysed in RIPA buffer (1% IGEPAL CA‐630, 0.1% SDS, and 0.5% sodium deoxycholate in PBS) supplemented with Phosphatase Inhibitor Cocktail 2 (Sigma, P5726, St. Louis, MO, USA) and Cocktail 3 (Sigma, P0044) and Complete Protease Inhibitor Cocktail (Sigma, 1 186 145 001). Lysate was sonicated using Sonics Vibra cell VCX130 (Sonics & Materials Inc., Newtown, CT, USA) (30 s; pulses 1 s on, 1 s off; 40% amplitude) then centrifuged at 12000 rcf for 10 min at 4 °C. Protein content was determined using Pierce BCA Protein Assay Kit (ThermoScientific, Waltham, MA, USA). Lysates were adjusted with Laemmli loading buffer (5×: 60 mm Tris/Cl pH 6.8, 10% glycerol, 1% SDS, 0.05% Bromophenol Blue, 1% beta‐mercaptoethanol). Immunoblotting was performed as described elsewhere [98]. The following primary antibodies were used: MCAD (Abcam, AB92461, Cambridge, MA, USA) and HSP90 (Cell Signaling, 4874S, Danvers, MA, USA).

### *In vitro* experiments

HepG2 cells were cultured in maintenance medium (Table 1), unless stated otherwise, and kept at 37 °C and 5% CO_2_. For the experiments, 2‐4 × 10^6^ cells were seeded in 100 mm plates and kept for 24–48 h in maintenance medium until reaching ~70% confluency. In the day of the experiment, cells were washed 2 times with PBS (Gibco), and the maintenance medium was replaced by the condition medium: (I) Palmitate, low‐glucose, or (II) Palmitate, no‐glucose (Table 1). After 24 h, the cells were washed twice with ice‐cold PBS (Gibco) and harvested for further analysis.

**Table 1:** Composition of the media used for *in vitro* experiments.

Medium components	Maintenance	Palmitate, low glucose	Palmitate, no glucose
DMEM^a^	P04‐01500	P04‐01500	P04‐01548S1
FBS^b^	10%	10%	10%
Glucose	5 mm	5 mm	–
Pyruvate	1 mm	1 mm	–
Glutamine	3 mm	3 mm	–
L‐Carnitine	–	2 mm	2 mm
BSA‐bound Palmitate	–	0.5 mm	0.5 mm

^a^ Dulbecco's Modified Eagle Medium (PAN Biotech™).

^b^ Fetal Bovine Serum (Gibco).

For label incorporation experiments, pantothenate‐free DMEM (PAN Biotech™) was supplemented with 16 μM of ^13^C_3_‐^15^N‐pantothenate (Sigma, 705 837‐5MG), a concentration equal to that of unlabelled pantothenate in regular DMEM. Moreover, dialysed FBS was used instead of normal FCS (Fisher scientific, sh3007902, Waltham, MA, USA). The concentrations of glucose, pyruvate, glutamine, palmitate, and L‐carnitine were the same as Palmitate low‐glucose medium (Table 1).

In each individual experiment, WT and 3 MCAD‐KO clones were tested in parallel, and each condition had 5 technical replicates (5 parallel cultures). All the experiments were performed at least 3 times (independent experiments). In total, for each cell line, between 15 and 18 separate cell cultures were assayed in 3–4 rounds of measurements.

### Animal experiments

A previously generated MCAD‐KO mouse model on a C57BL/6NTac & 129P2/OlaHsd mixed background [49] was obtained from the original authors and backcrossed on a C57BL/6J pure background [99] (C57BL/6J background mice supplied by The Jackson Laboratory, Bar Harbor, ME, USA; stock no. 000664). Male MCAD‐KO and wild‐type (WT) littermate mice on a C57BL/6J background were kept in the housing facility under temperature‐ (21 °C) and light‐controlled (12 h light) conditions and had free access to food and drinking water. In the first experiment, 8‐week‐old WT and MCAD‐KO mice were divided into two groups: (1) fed and (2) 14 h‐fasted. Both groups were fed with commercially available laboratory chow diet (V1554‐703, Ssniff). For the fasted group, on the day of the experiment, WT and MCAD‐KO mice were transferred to a new cage without food, but with free access to water. The animals were overnight fasted for 14 h at 21 °C and sacrificed. In a second experiment, 8‐week‐old WT and MCAD‐KO mice were exposed to a third condition, (3) 14 h‐fasted and cold exposed [99]. For condition (3), prior to the start of the experiment, at the age of 4 weeks, mice were fed with a chow‐like semi‐synthetic diet (D12450B, Research Diet Services) for 4 weeks. On the experiment day, 8‐week WT and MCAD‐KO mice were 14 h‐overnight fasted at 21 °C, then transferred to a 4 °C environment for 4 h, also fasted (total of 18 h fasting). For all the experimental conditions (1. Fed; 2. Fasted; 3. Fasted and cold exposed), mice were terminated via cardiac puncture under isoflurane anaesthesia, and the liver was collected for biochemical analysis.

### Ethics statement

All animal experiments were approved by the Institutional Animal Care and Use Committee of the University of Groningen (Groningen, The Netherlands) with study identification codes IvD 15 167‐01‐03 (simultaneous fasting and cold exposure) and IvD 15 244–02‐063 (fed and fasted conditions). All experiments are in line with the Guide for the Care and Use of Laboratory Animals.

### Acyl‐CoA and acylcarnitine profiling

#### Cell samples

Sample preparation was performed using a two‐step approach according to the approach developed by Bligh and Dyer [100, 101]. For acyl‐CoA and acylcarnitine profiling, an Acquity UPLC system from Waters (Milford, MA, USA) coupled to an AB Sciex QTRAP 6500 mass spectrometer equipped with Turbo V source (Concord, ON, Canada) was utilised. Both acyl‐CoA and acylcarnitine analyses were conducted using positive electrospray ionisation (ESI) in scheduled multiple reaction monitoring (sMRM) mode. The acyl‐CoA profiling was performed using HILIC‐MS/MS conditions according to the previously published study [31], employing a SeQuant® ZIC®‐cHILIC HPLC (100 × 2.1 mm), with a particle size of 3.0 μm and a pore size of 100 Å (Merck, Darmstadt, Germany). The detailed explanation can be obtained from the previous publication [31]. The acylcarnitine profiling was performed using a reversed‐phase liquid chromatography–tandem mass spectrometry (RPLC–MS/MS) method using the AccQ‐Tag™ Ultra C18 column with dimensions of 2.1 × 100 mm and a particle size of 1.7 μm from Waters (Milford, MA, USA).

Liquid chromatography‐mass spectrometry (LC–MS) grade solvents, namely acetonitrile, methanol, isopropanol and chloroform, were procured from BioSolve BV (Valkenswaard, The Netherlands). Ammonium acetate (≥99%) was purchased from Sigma‐Aldrich (St. Louis, MO, USA). Pentadecanoyl‐CoA (C15:0‐CoA) and heptadecanoyl‐CoA (C17:0‐CoA) in ammonium salt form were purchased from Avanti Polar Lipids (Alabaster, AL, USA). Acetyl‐1,2‐^13^C_2_‐CoA (C2:0(^13^C_2_)‐CoA) and *n*‐heptanoyl‐CoA (C7:0‐CoA) as lithium salts were obtained from Sigma‐Aldrich (St. Louis, MO, USA). Acylcarnitines deuterium labelled standards, L‐carnitine‐d_3_, propionyl‐L‐carnitine‐d_3_, butyryl‐L‐carnitine‐d_3_, octanoyl‐L‐carnitine‐d_3_ and octadecanoyl‐L‐carnitine‐d_3_ as hydrochloride salts were purchased from CDN Isotopes (Pointe‐Claire, QC, Canada).

#### Acyl‐CoA and acylcarnitine extraction from HepG2 cells

HepG2 cells were washed twice in ice‐cold PBS (Gibco) and spun down at 1000 ***g*** for 3 min at 4 °C. The cell pellet was reconstituted in 220 μL 80% methanol, of which 120 μL were used for protein quantification using the BCA method. The remaining 100 μL was used for acyl‐CoA and acylcarnitine profile analyses (starting volume). Prior to extraction, a mixture of 10 μL of acyl‐CoA and acylcarnitine internal standards (Acetyl‐1,2‐^13^C_2_‐CoA, C7:0‐CoA, C15:0‐CoA, C17:0‐CoA, L‐carnitine‐d_3_, propionyl‐L‐carnitine‐d_3_, butyryl‐L‐carnitine‐d_3_, octanoyl‐L‐carnitine‐d_3_, and octadecanoyl‐L‐carnitine‐d_3_) was added to the study samples and vortexed for 1 min. Subsequently, 220 μL of methanol and 100 μL of water were added to each sample, followed by vortexing for 2 min and sonication for 3 min. To each sample, 320 μL of chloroform and 188 μL of water were added, vortexed for 2 min, and allowed to partition on ice for 10 min. The samples were then centrifuged for 15 min at 4 °C with a speed of 15 800 ***g*.** The resulting upper aqueous layer (450 μL) was transferred to a new 1.5 mL tube. Additionally, the lower organic layer (250 μL) was transferred to the same 1.5 mL tube. The combined solution was completely evaporated using a Labconco CentriVap vacuum concentrator located in Kansas City, MO, USA. Following evaporation, the samples were reconstituted in a mixture of methanol/water/isopropanol (1:1:1) to a final volume of 100 μL and injected into the LC–MS system.

#### Settings and configuration for RPLC–MS/MS for analysing acylcarnitines

For the separation of acylcarnitines by RPLC, the mobile phase A (MPA) consisted of 0.1% formic acid in water, while mobile phase B (MPB) contained 0.1% formic acid in acetonitrile. The injection volume was set at 5 μL, and the autosampler temperature was maintained at 10 °C, while the column temperature was set to 60 °C. A flow rate of 0.7 mL·min^−1^ was employed. The gradient elution profile spanning 11 min is provided in Table 2.

**Table 2:** Gradient elution profile for acylcarnitine profiling.

Time (min)	0	1.10	1.11	2.00	8.00	8.01	9.01	9.20	11.00
MPA (%)	95.0	95.0	89.0	89.0	30.0	0	0	95.0	95.0
MPB (%)	5.0	5.0	11.0	11.0	70.0	100.0	100.0	5.0	5.0

The QTRAP 6500 mass spectrometer was operated with the following parameters: the curtain gas (N2) pressure was set to 20 psi, and the collision gas (N2) was maintained at a medium level. For positive ion mode, the ion spray voltage was set at 4500 V. The source temperature was maintained at 350 °C, while the GS1 and GS2 pressures were set to 80 and 70 psi, respectively. Data acquisition for targeted analysis was performed using scheduled multiple reaction monitoring (sMRM), with a target scan time of 0.1 s. Transitions and retention times are provided in Table 3.

**Table 3:** MRM transitions of acylcarnitines for LC–MS/MS analysis. IS, internal standard.

Acylcarnitines	Q1 (parent ion)	Q3 (parent ion)	Declustering potential (V)	Collision energy (eV)	Retention time (min)
Free carnitine (C0)	162.2	85.1	70	25	0.34
Acetylcarnitine (C2:0)	204.2	85.1	70	10	0.36
C4:0^a^	232.3	85.1	70	30	1.31
Hexanoylcarnitine (C6:0)	260.3	85.1	70	30	3.4
Octanoylcarnitine (C8:0)	288.3	85.1	70	30	4.72
Decanoylcarnitine (C10:0)	316.3	85.1	70	30	5.72
Lauroylcarnitine (C12:0)	344.4	85.1	70	30	6.61
Myristoylcarnitine (C14:0)	372.5	85.1	70	30	7.42
Palmitoylcarnitine (C16:0)	400.5	85.1	70	30	8.16
L‐Carnitine‐d3 (IS)	165.2	85.1	70	30	0.34
Propionyl‐L‐carnitine‐d3	221.1	85.1	70	30	0.54
Butyryl‐L‐carnitine‐d3 (IS)	235.3	85.1	70	30	1.34
Octanoyl‐L‐carnitine‐d3 (IS)	291.3	85.1	70	30	4.71
Octadecanoyl‐L‐carnitine‐d3 (IS)	431.6	85.1	70	30	8.42

^a^ There are multiple isomers possible for C4:0 carnitine, hence it is denoted by the carbon number.

The peak integration for both acyl‐CoA and acylcarnitines was performed using AB Sciex OS (version 2.1.6, AB SCIEX, Concord, ON, Canada).

#### Murine liver and blood samples

Acylcarnitine levels were extracted and measured according to Derks et al [16], using an API 3000 LC‐MS/MS equipped with a Turbo ion spray source (Applied Biosystems/MDS Sciex, Ontario, Canada).

### Total CoA measurements

Cell pellets and liver samples for total CoA measurements were prepared as described elsewhere [102] and detailed below.

#### Murine liver samples

Briefly, 500 μL MilliQ H_2_O was added to 25–100 mg liver tissue (adjusted to a final concentration of 0.2 mg liver·μL^−1^), and the tissue was lysed using a tissue homogeniser (Precellys, Bertin Instruments) (6000 rpm, 15 s, 2 times). Lysates were centrifuged at 20 000 ***g*** for 15 min at 4 °C. In a new tube, 10 μL Tris (2‐carboxyethyl) phosphine hydrochloride (50 mm) was added to 50 μL supernatant vortex and incubated for 15 min at room temperature. Next, 40 μL saturated ammonium sulphate was added, and samples were centrifuged 20 000 ***g*** for 15 min at 4 °C. The clear supernatant (50 μL) was then derivatized with 45 μL SBD‐F (ammonium 7‐flurobenzo‐2‐oxa‐1,3‐doazole‐4‐sulfonate; 1 mg·mL^−1^ in borax buffer: 0.1 M containing 1 mm EDTA, pH 9.5) plus 5 μL ammonia solution (12,5% v/v), and incubated shaking at 500 rpm at 60 °C for 1 h (protected from light). Lastly, samples were spun down, and the supernatant was measured via HPLC as described elsewhere [102].

#### Cell samples

The cell pellet was reconstituted in 600 μL MilliQ H_2_O and the lysate was sonicated using a Sonics Vibra cell VCX130 (Sonics & Materials Inc.) (25 s, 50% amplitude, 2 times). Lysates were centrifuged at 18 400 ***g*** for 15 min at 4 °C. In a new tube, 80 μL Tris (2‐carboxyethyl)phosphine hydrochloride (10 mm) was added to 400 μL supernatant and incubated for 15 min at room temperature. Next, samples were spun down (18 400 ***g***, 15 min, 4 °C). In a new tube, 40 μL ammonia solution (12.5% v/v) was added to 400 μL supernatant and, incubated shaking at 500 rpm at 60 °C for 60 min. Lastly, samples were dried using a SpeedVac (Eppendorf, Hamburg, Germany) and reconstituted in 100 μL ice‐cold 80% methanol. CoA was measured using HILIC‐MS/MS as detailed by Singh *et al*. [31].

### RNA extraction and quantitative PCR

RNA was isolated using the RNeasy® Plus Universal Mini Kit (Qiagen, 73 404, Germantown, CA, USA). qPCR was performed using FastStart Universal Sybr Green (Roche, 0413914001) on QuantStudio™ 7 (Applied Biosystems, Waltham, MA, USA). The thermal cycling consisted of 10 min hold at 95 °C, followed by 40 cycles of 15 s at 95 °C, 30 s at 60 °C, and 30 s at 72 °C. Forward and reverse primers for human and murine genes are listed in Tables S5 and S6, respectively. The Ct values were expressed relative to the *YWHAZ* gene (HepG2 cells) and or the *36B4* gene (mouse) and normalised to the average expression level in the WT controls.

## Conflict of interest

This paper and its authors have no conflict of interest with any of the other research groups, or organisations.

## Author contributions

CO, LAK, MS, ACH, TH, and BMB designed the experiments and simulations. CO, LAK, and MS performed the experiments, generated and interpreted the data, and wrote the manuscript. MS with the support of AD, VC, and GLFK. developed, optimised and performed the HILIC‐MS protocol used in this work. CO performed all the computational simulations. MLM and AG supported with the *in vitro* experiments and qPCR analyses. MvdZ performed the HPLC analysis under the supervision of OCMS, ACMFM and LAK designed and conducted the animal experiments. NCAH and LAK carried out the overexpression experiments under the supervision of BvdS, HS, DJR, and OCMS critically reviewed the manuscript at various junctures, providing valuable input on data analysis and interpretation. ACH, TH, and BMB supervised the project and edited the draft manuscript. All authors commented on the text.

## Supporting information


**Fig. S1.** Partial MCAD reintroduction in MCAD‐KO HepG2 cells partially restores phenotype.
**Fig. S2.** Acylcarnitine profile of cells incubated for 24 h in *palmitate no‐glucose* medium.
**Fig. S3.** Acyl‐CoA profile of cells incubated for 24 h in *palmitate no‐glucose* medium.
**Fig. S4.** Heat maps of acyl‐CoAs and acylcarnitines showing differences between MCAD‐KO clones and WT incubated in *Palmitate no‐glucose* medium.
**Fig. S5.** Acyl‐CoA and acylcarnitine profiles of cells incubated for 24 h in *palmitate low‐glucose* medium.
**Fig. S6.** Heat maps of acyl‐CoAs and acylcarnitines showing differences between MCAD‐KO clones and WT incubated in *Palmitate low‐glucose* medium.
**Fig. S7.** Acyl‐CoA and acylcarnitine profiles of WT and MCAD‐KO HepG2 cells grown under two conditions.
**Fig. S8.** Change in free and total CoA over 24 h of palmitate/L‐carnitine exposure.
**Fig. S9.** Body temperature, blood glucose, and acylcarnitines of mice.
**Fig. S10.** PANK isoform expression under nutrient stress.
**Fig. S11.** Gene expression data from MCAD‐KO HepG2 cells on *Palmitate no‐glucose medium*.
**Fig. S12.** Heat map of gene expression data showing differences between MCAD‐KO clones and WT incubated in *Palmitate no‐glucose* medium.
**Fig. S13.** Gene expression data from MCAD‐KO HepG2 cells on *Palmitate low‐glucose* medium.
**Fig. S14.** Heat map of gene expression data showing differences between MCAD‐KO clones and WT incubated in *Palmitate low‐glucose* medium.
**Table S1.** Statistical significance of differences in CoASH and total CoA levels between HepG2 cells divided in T0H and T24 groups.
**Table S2.** Statistical significance of differences in CoASH and total CoA levels between MCAD‐KO clones and WT in T0H and T24H.
**Table S3.** Statistical significance of differences in percentage label incorporation into the CoASH fraction and into the total CoA pool over the course of 24 h between T0H and T24 groups.
**Table S4.** Statistical significance of differences in percentage label incorporation into the CoASH fraction and into the total CoA pool over the course of 24 h between MCAD‐KO clones and WT.
**Table S5.** List of human primer sequences used in RT‐qPCR.
**Table S6.** List of murine primer sequences used in RT‐qPCR.

## Data Availability

The data that support the findings of this study are openly available on the FAIRDOM Hub at https://fairdomhub.org/studies/1363/snapshots/3.
