# Supplementary material for: Energetic stress in combination with impaired fatty acid oxidation induces sequestration of CoA and adaptation of CoA metabolism
Source: FEBS J. 2026 Feb 7;293(12):3565–87. doi: 10.1111/febs.70442 (PMC13278353; doi:10.1111/febs.70442)
Supplement: Supplementary file 1 — Fig. S1. Partial MCAD reintroduction in MCAD‐KO HepG2 cells partially restores phenotype. Fig. S2. Acylcarnitine profile of cells incubated for 24 h in palmitate no‐glucose medium. Fig. S3. Acyl‐CoA profile of cells incubated for 24 h in palmitate no‐glucose medium. Fig. S4. Heat maps of acyl‐CoAs and acylcarnitines showing differences between MCAD‐KO clones and WT incubated in Palmitate no‐glucose medium. Fig. S5. Acyl‐CoA and acylcarnitine profiles of cells incubated for 24 h in palmitate low‐glucose medium. Fig. S6. Heat maps of acyl‐CoAs and acylcarnitines showing differences between MCAD‐KO clones and WT incubated in Palmitate low‐glucose medium. Fig. S7. Acyl‐CoA and acylcarnitine profiles of WT and MCAD‐KO HepG2 cells grown under two conditions. Fig. S8. Change in free and total CoA over 24 h of palmitate/L‐carnitine exposure. Fig. S9. Body temperature, blood glucose, and acylcarnitines of mice. Fig. S10. PANK isoform expression under nutrient stress. Fig. S11. Gene expression data from MCAD‐KO HepG2 cells on Palmitate no‐glucose medium. Fig. S12. Heat map of gene expression data showing differences between MCAD‐KO clones and WT incubated in Palmitate no‐glucose medium. Fig. S13. Gene expression data from MCAD‐KO HepG2 cells on Palmitate low‐glucose medium. Fig. S14. Heat map of gene expression data showing differences between MCAD‐KO clones and WT incubated in Palmitate low‐glucose medium. Table S1. Statistical significance of differences in CoASH and total CoA levels between HepG2 cells divided in T0H and T24 groups. Table S2. Statistical significance of differences in CoASH and total CoA levels between MCAD‐KO clones and WT in T0H and T24H. Table S3. Statistical significance of differences in percentage label incorporation into the CoASH fraction and into the total CoA pool over the course of 24 h between T0H and T24 groups. Table S4. Statistical significance of differences in percentage label incorporation into the CoASH fraction and into the total CoA po [file FEBS-293-3565-s001.pdf]

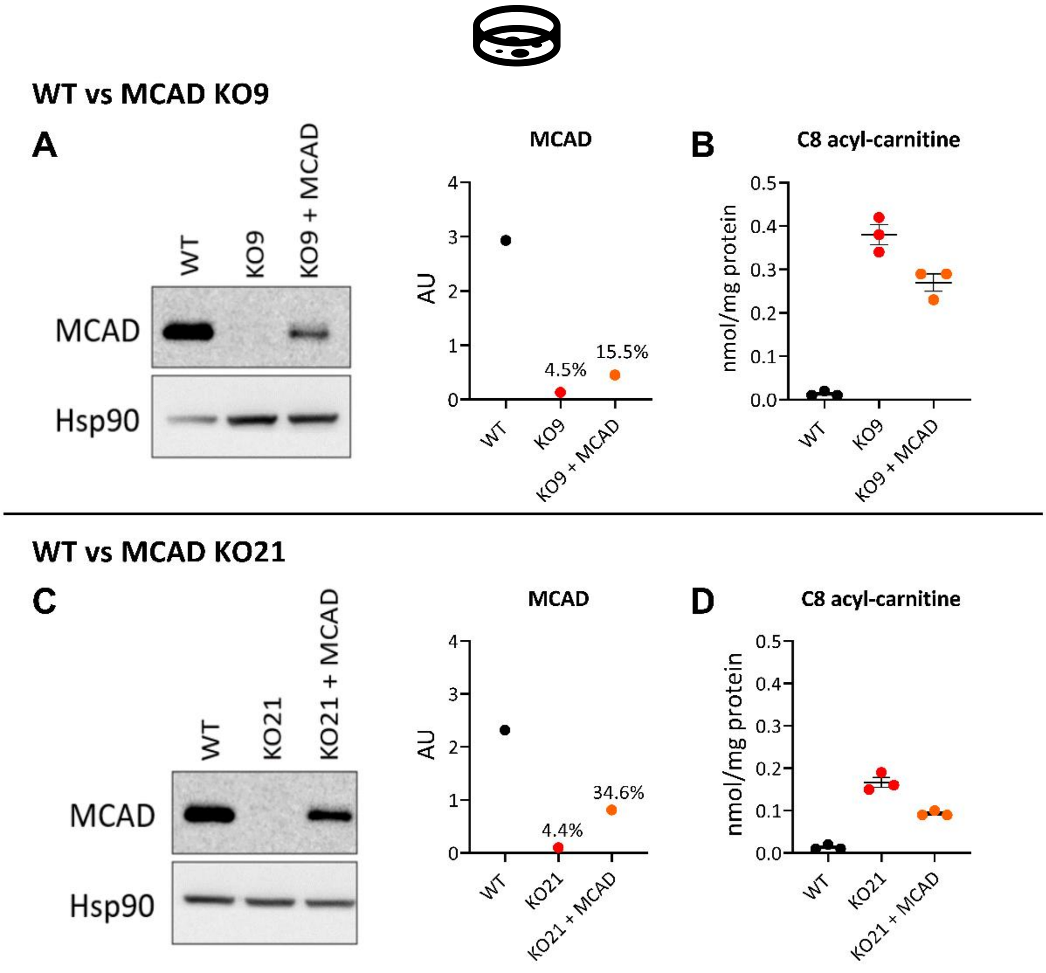

Figure S1. Partial MCAD reintroduction in MCAD-KO HepG2 cells partially restores phenotype. A. Representative immunoblot image and respective quantification of MCAD protein in WT, MCAD-KO clone KO9 cells, and KO cells overexpressing MCAD (KO9 + MCAD); HSP90 (heat shock protein) was used as loading control. **B.** Intracellular level of C8-acylcarnitine; n=3 technical replicates. **C.** Representative immunoblot image and respective quantification of MCAD protein in WT, MCAD-KO clone KO21 cells, and KO cells overexpressing MCAD (KO21 + MCAD). **D.** Intracellular level of C8-acylcarnitine; n=3 technical replicates.

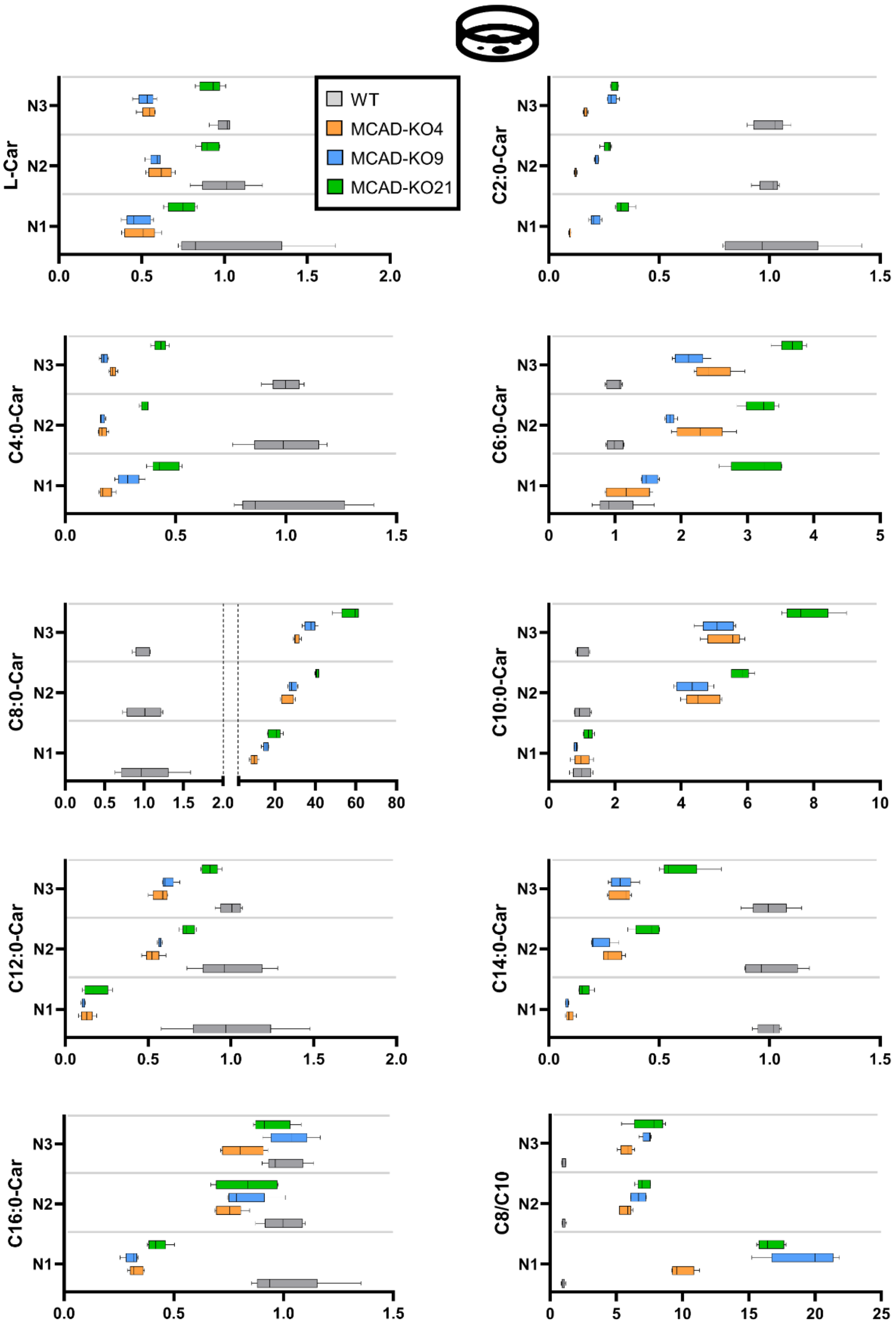

Figure S2. Acylcarnitine profile of cells incubated for 24h in *palmitate no-glucose* medium. Acylcarnitine levels relative to mean WT levels within each technical repeat of the assay. Detailed data set of Fig. S2B. N1, N2, and N3 represent biological replicates. Each box contains 3-5 technical replicates. Boxes indicate the (interquartile) range and median relative to the mean WT value within each biological repeat. **Statistics are displayed in Figure S4**.

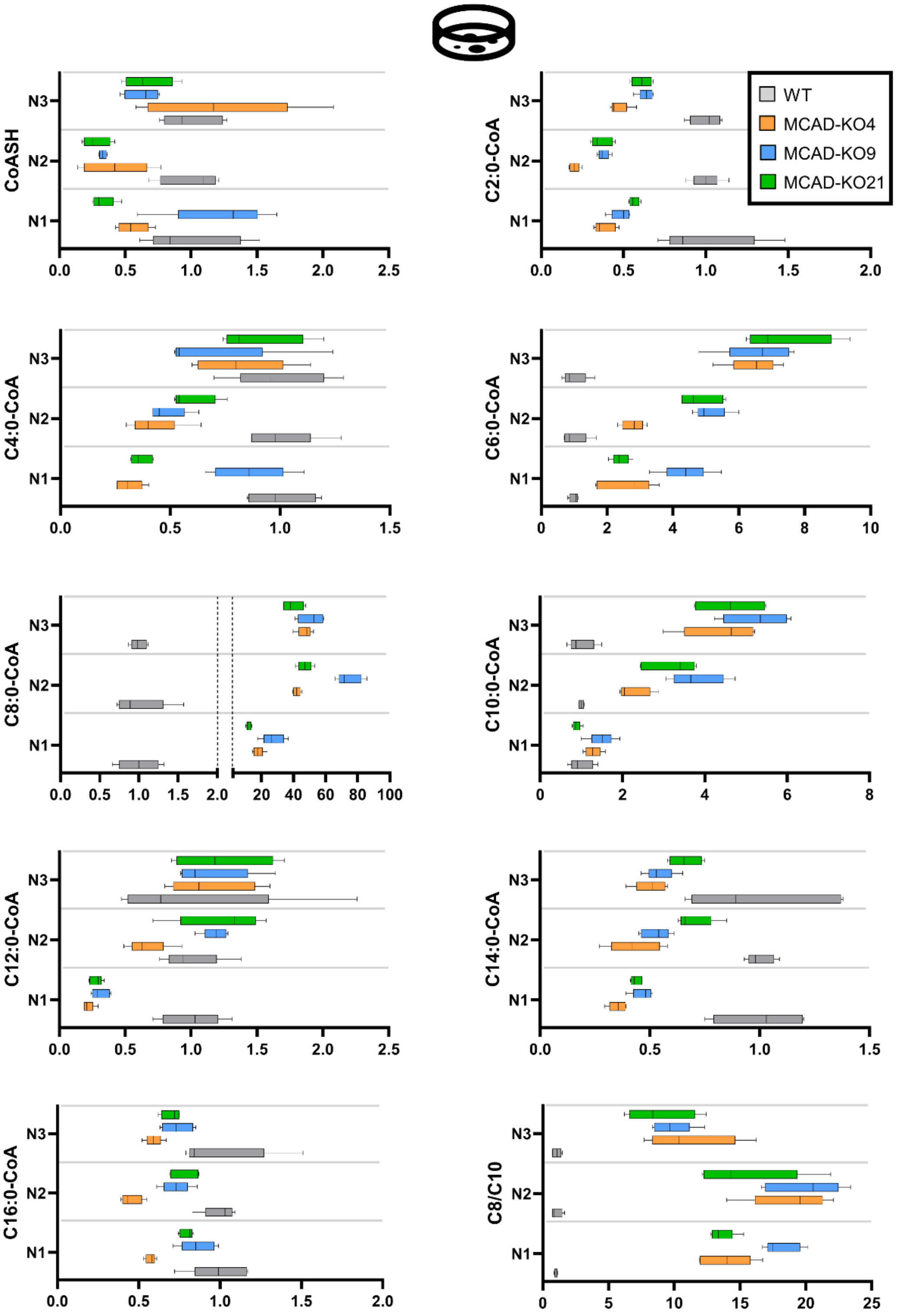

Figure S3. Acyl-CoA profile of cells incubated for 24h in *palmitate no-glucose* medium. Acyl-CoA levels relative to mean WT levels in HepG2 cells. N1, N2, and N3 represent biological replicates. Detailed data set of Fig. S2C. Each box contains 3-5 technical replicates. Boxes indicate the (interquartile) range and median relative to the mean WT value within each biological repeat. **Statistics are displayed in Figure S4**.

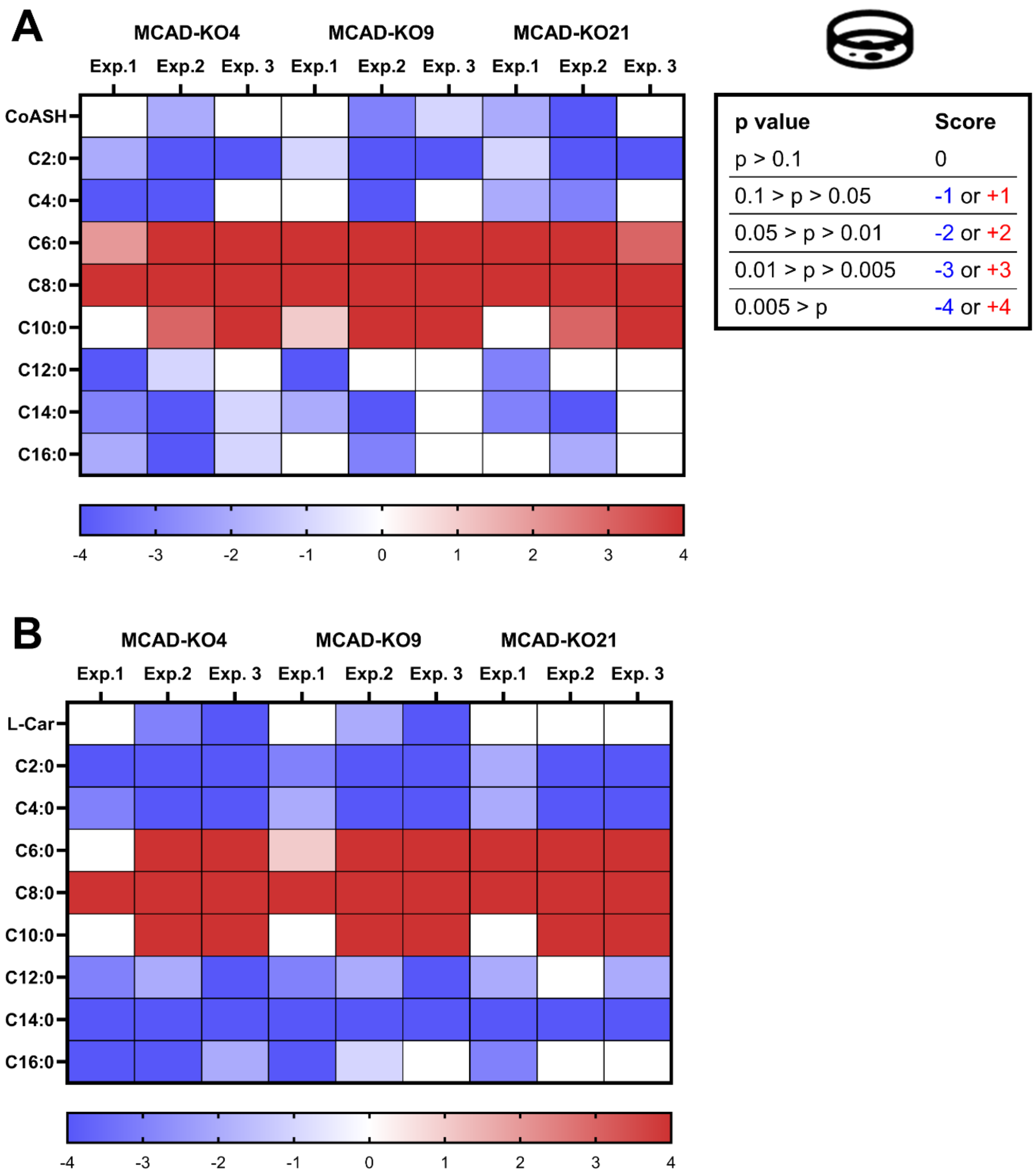

Figure S4. Heat maps of acyl-CoAs and acylcarnitines showing differences between MCAD-KO clones and WT incubated in *Palmitate no-glucose* medium. A. Acyl-CoA and **B.** acylcarnitine profiles. One-way Brown-Forsythe ANOVA adjusted by Dunnett's T3 multiple comparisons test was performed per individual experiment (n=3) on the data displayed in Fig. 2C and detailed in Fig. S2 and S3. These were therefore values relative to the WT mean within each experiment. Each experiment consisted of 4-5 cultures. The scores (-4 to +4) in the heat maps represent the p values as follow, 0: p > 0.1, +1 or -1: 0.1 > p > 0.05, +2 or -2: 0.05 > p > 0.01, +3 or -3: 0.01 > p > 0.005 , +4 or -4: p < 0.005. Red cells (and positive scores) indicate cases where the KO had a higher concentration relative to the WT, while blue cells (negative scores) indicate decreases. White cells indicate no significant changes (p>0.1).

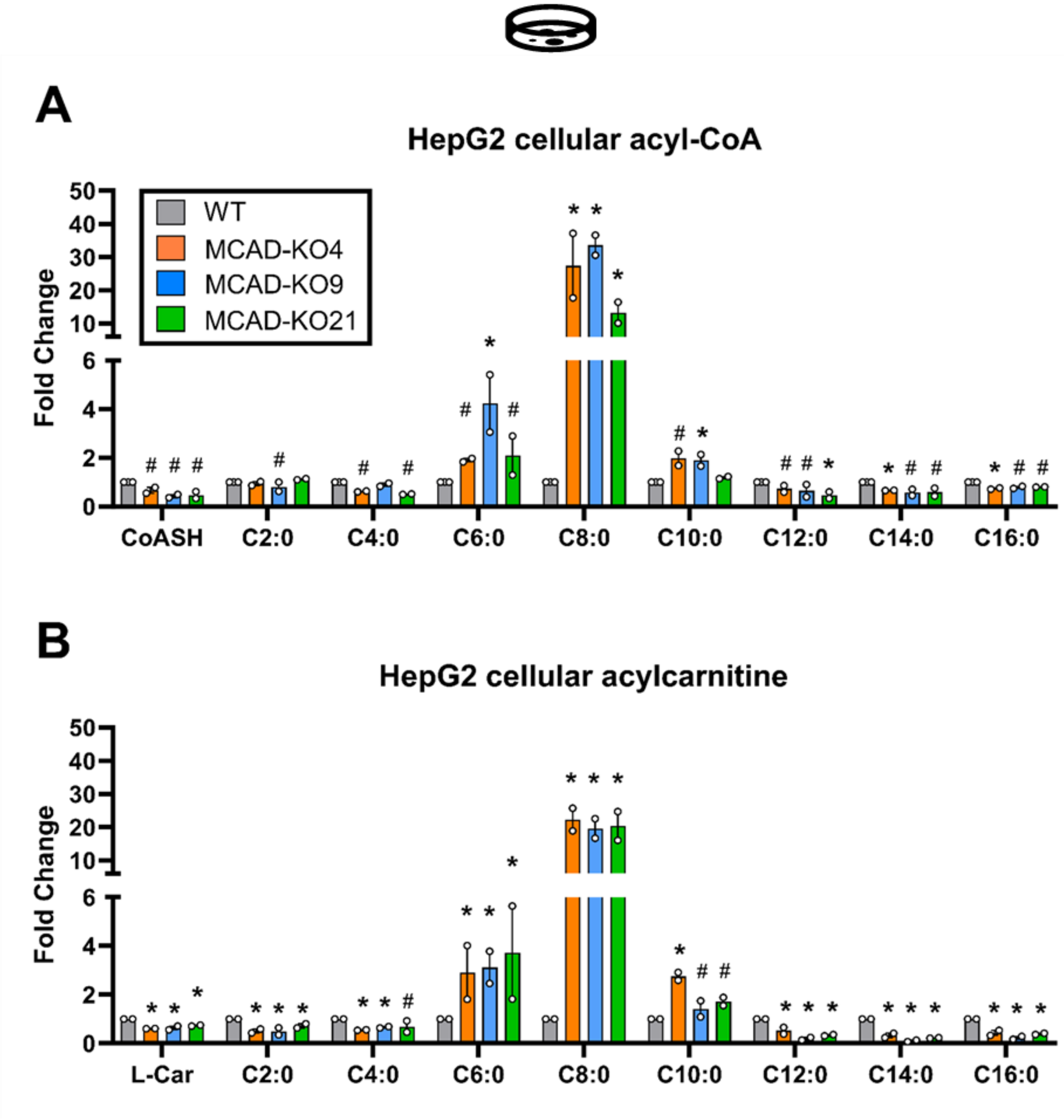

Figure S5. Acyl-CoA and acylcarnitine profiles of cells incubated for 24h in *palmitate low-glucose* medium. A. Acyl-CoA and **B.** acylcarnitine levels relative to mean WT levels in HepG2 cells. Each data point represents the average of an independent experiment (2 experiments), which consisted of 4-5 technical replicates (cell cultures); ± standard error of the mean (SEM); *p < 0.05 in 2 out of 2 experiments, #p < 0.05 in 1 out of 2 experiments. **Statistics are displayed in Figure S6**.

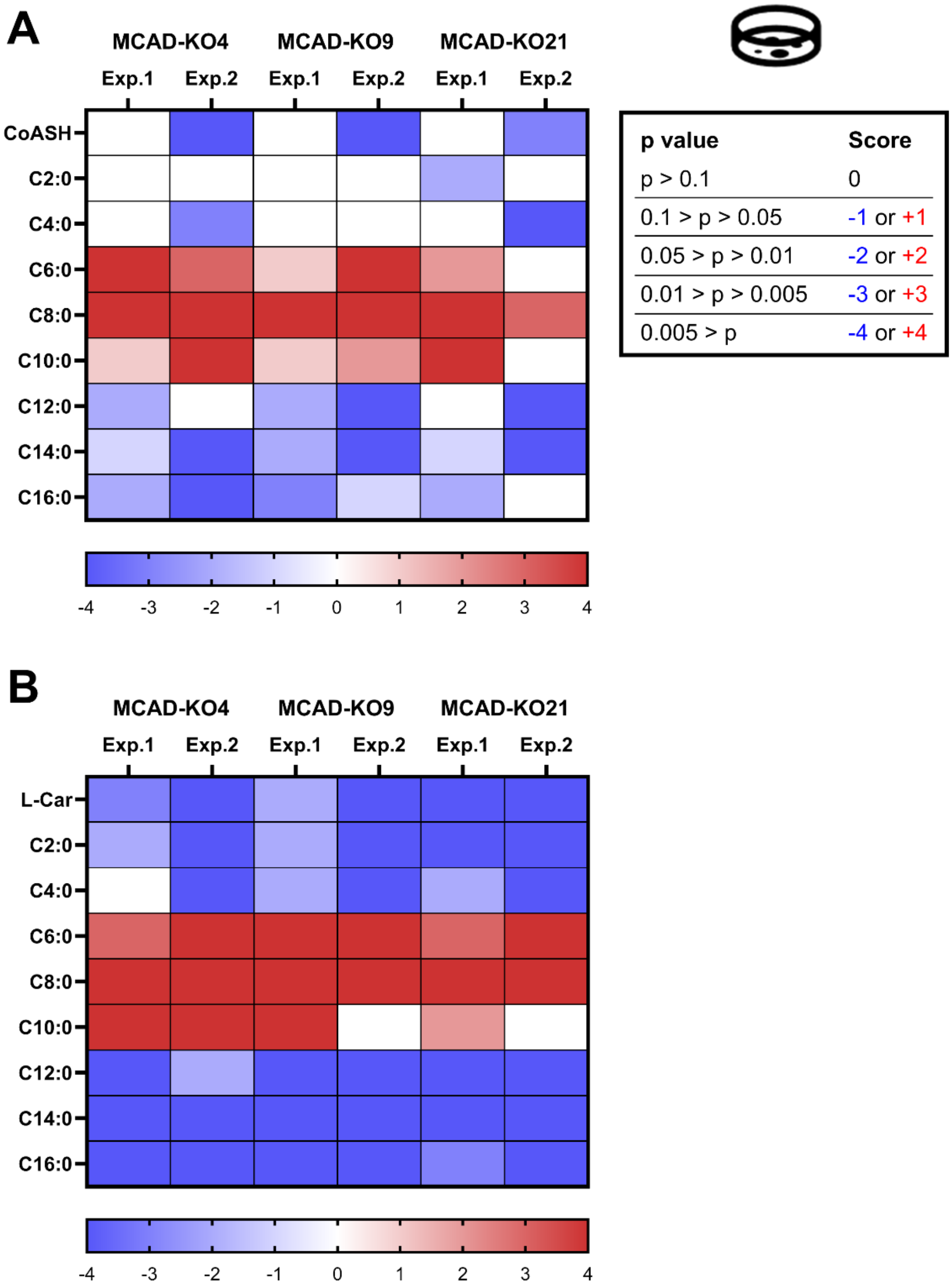

Figure S6. Heat maps of acyl-CoAs and acylcarnitines showing differences between MCAD-KO clones and WT incubated in *Palmitate low-glucose* medium. A. Acyl-CoA and **B.** acylcarnitine profiles. One-way Brown-Forsythe ANOVA adjusted by Dunnett's T3 multiple comparisons test was performed per individual experiment (n=2) on the data displayed in Fig. S5. These were therefore values relative to the WT mean within each experiment. Each experiment consisted of 4-5 cultures. The scores (-4 to +4) in the heat maps represent the p values as follow, 0: $p > 0.1$, +1 or -1: $0.1 > p > 0.05$, +2 or -2: $0.05 > p > 0.01$, +3 or -3: $0.01 > p > 0.005$, +4 or -4: $p < 0.005$. Red cells (and positive scores) indicate cases where the KO had a higher concentration relative to the WT, while blue cells (negative scores) indicate decreases. White cells indicate no significant changes ($p>0.1$).

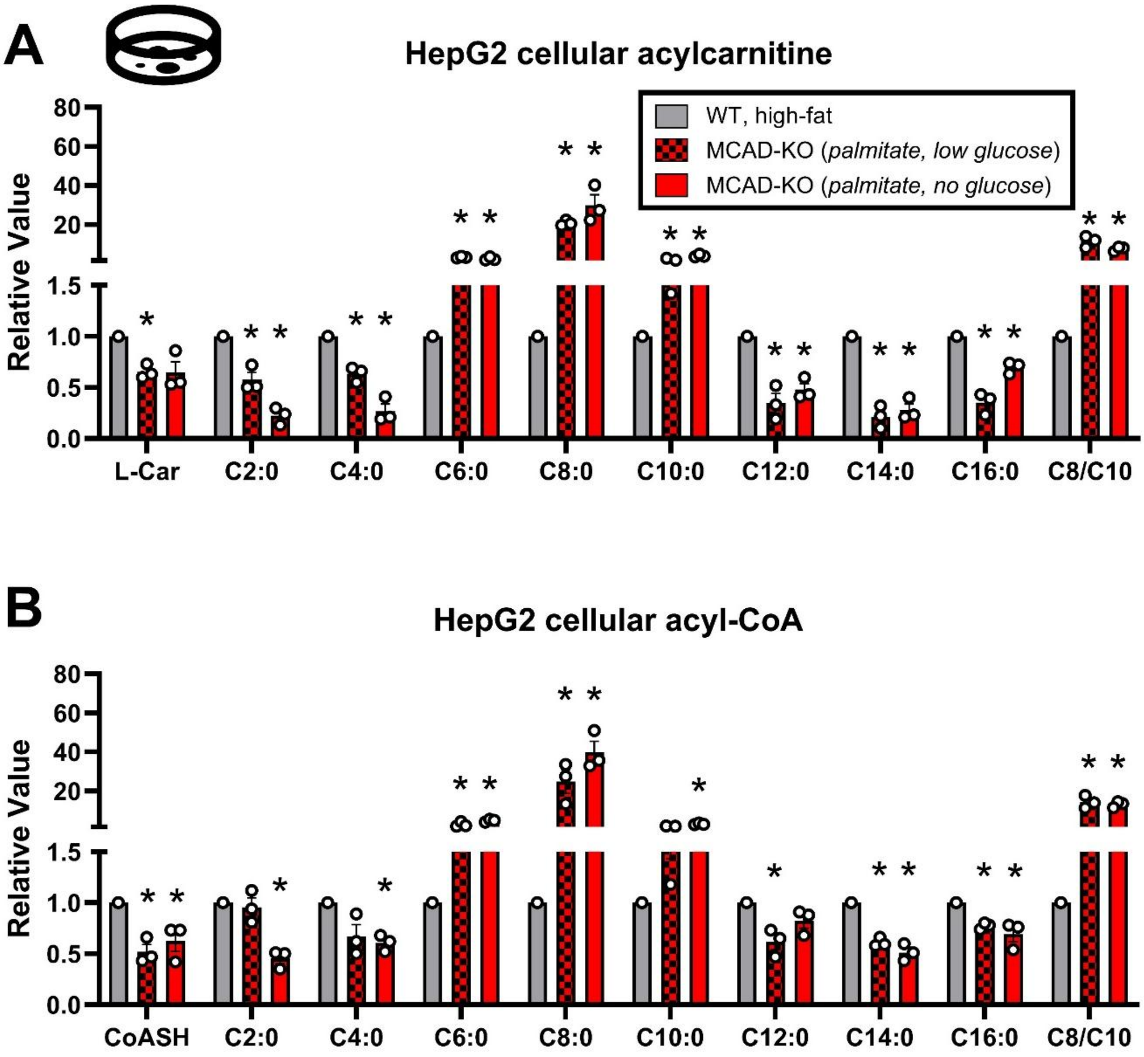

Figure S7. Acyl-CoA and acylcarnitine profiles of WT and MCAD-KO HepG2 cells grown under two conditions. Representative acyl-CoA and -carnitine accumulations relative to WT in HepG2 cells. Three KO-clones (KO4, KO9, KO21) and one WT cell line were incubated for 24h in two conditions: (I) palmitate low-glucose medium, and (II) palmitate no-glucose medium. Individual data points indicate the mean results for one cell line. The results consist of biological replicates (n=3-5) and technical replicates (n=3). The median and range are shown by the bar and error bars. **A.** Acylcarnitines. **B.** Acyl-CoA. Full data set in available online (link). * Statistically significant differences ($p < 0.1$) half or more of the biological replicates. Statistical significance per experiment in **Figures S4 and S6.**

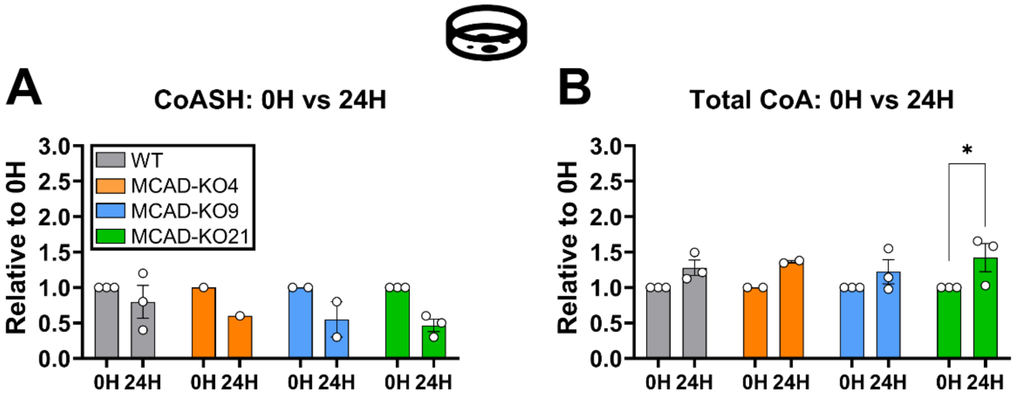

Figure S8. Change in free and total CoA over 24 hours of palmitate/L-carnitine exposure. Cells cultured for 24 hours in maintenance medium supplemented with 0.5 mM palmitate and 2.0 mM L-carnitine, in which non-labelled pantothenate was replaced by 4 mg/L stable-isotope-labelled pantothenate ($^{13}C_3$-^{15}N-VitB5). **A & B.** CoASH and total CoA relative to T0H in the same experiment (n=3; 3 independent experiments). Each individual experiment consisted of 4-5 technical replicates i.e., simultaneous cell cultures; Error bars represent ± standard error of the mean (SEM). **Statistics in Tables S1. Data from same experiment as in Fig. 3.**

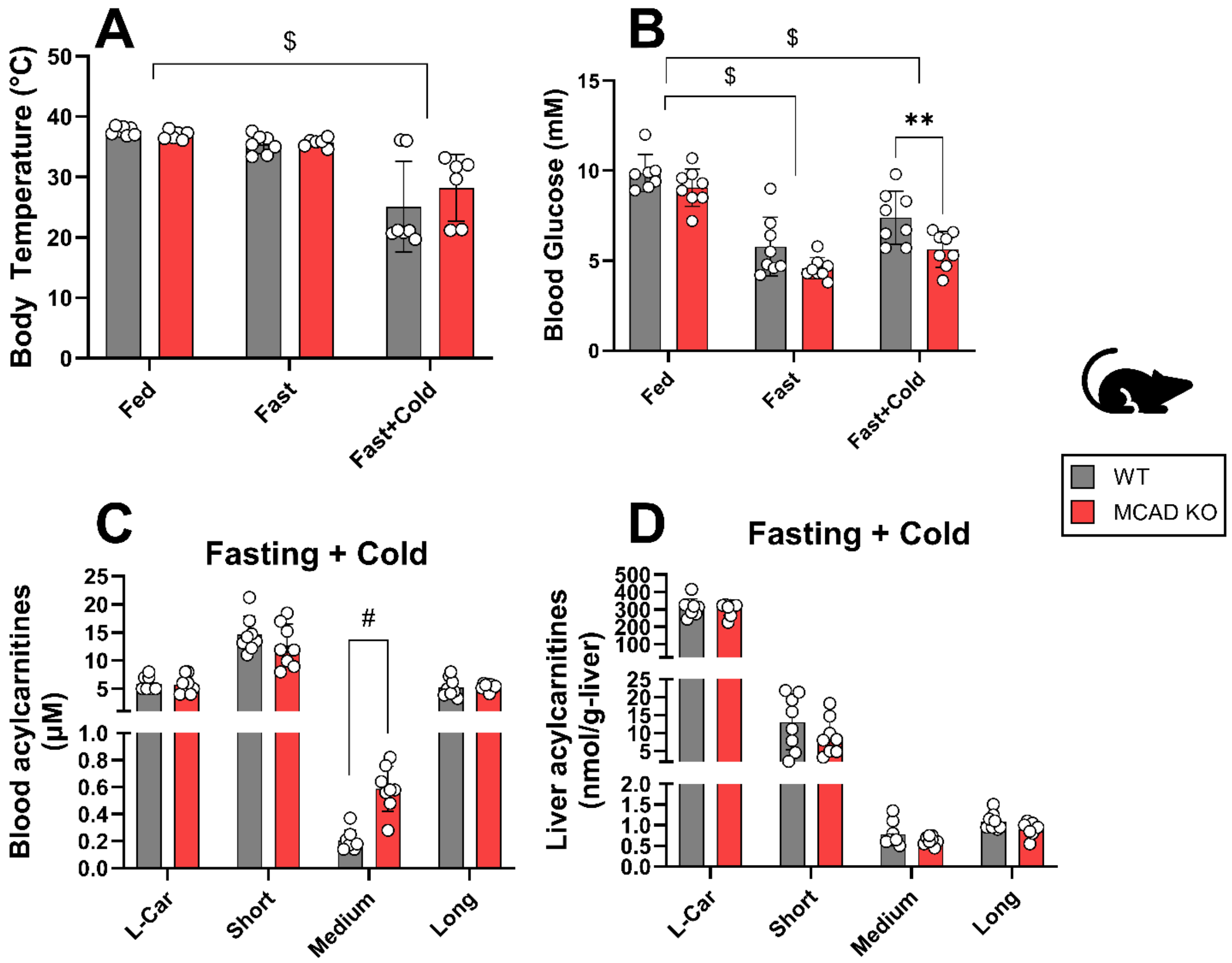

Figure S9. Body temperature, blood glucose, and acylcarnitines of mice. WT and MCAD-KO mice in a fed, fasted, and fasted + cold-exposed state **A.** Body temperature. **B**. Blood glucose. **C.** Blood acylcarnitines after fasting + cold exposure. **D.** Liver acylcarnitines after fasting + cold exposure. Acylcarnitines grouped into L-car (free, C0), short (C2-C5), medium (C6-C10), and long (C12-C18). ** $p < 0.01$ when comparing WT and MCAD-KO within a condition using a two-way ANOVA with Šídák's multiple comparisons test. \$ $p < 0.01$ for WT and MCAD-KO when comparing conditions within a genotype, also using two-way ANOVA with Šídák's multiple comparisons. # $p < 0.05$ using unpaired t-test.

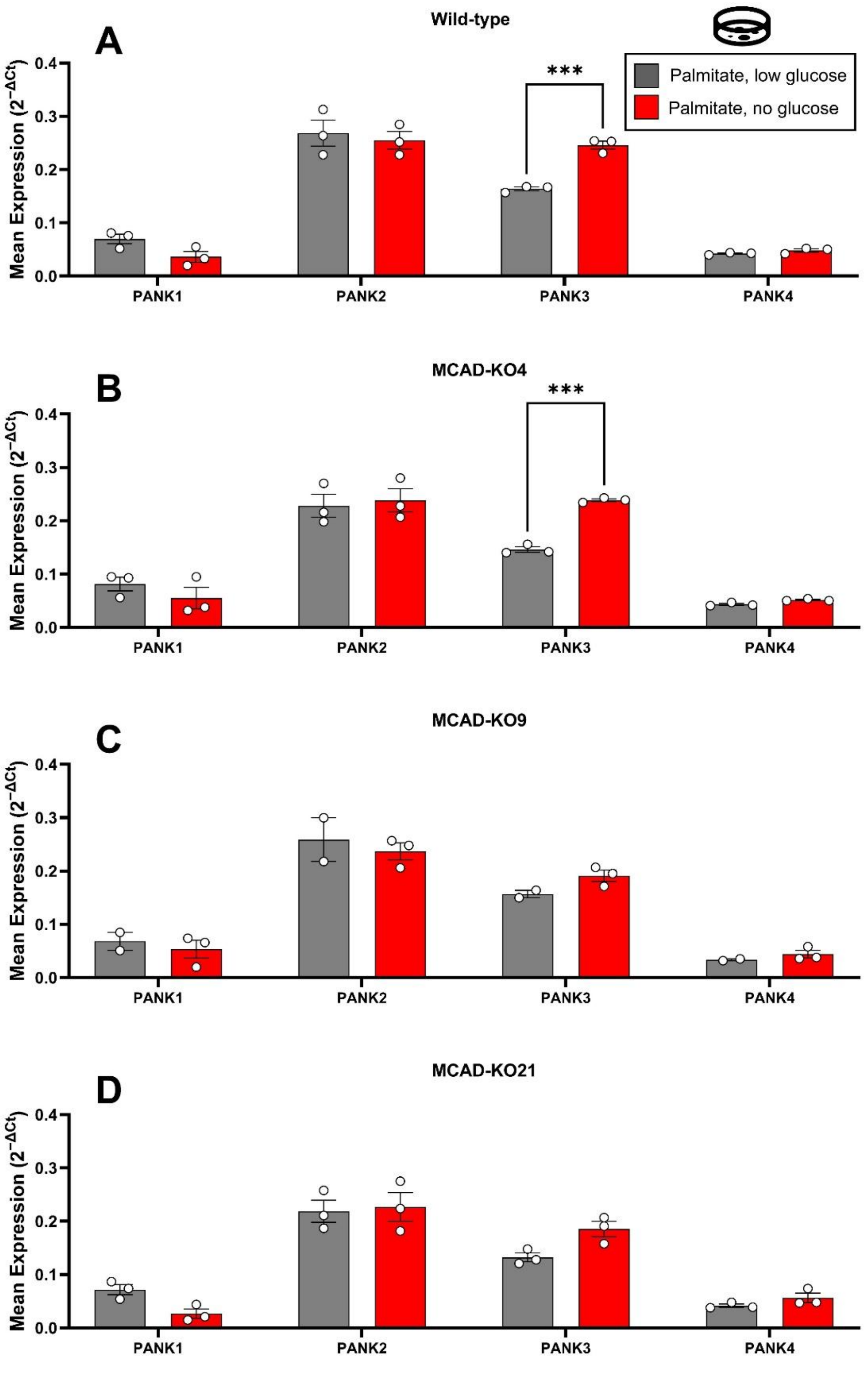

Figure S10. PANK isoform expression under nutrient stress. PANK expression in HepG2 cells in palmitate/low-glucose and palmitate/no-glucose medium, respectively. qPCR measurements normalised to the expression of a housekeeping gene (YWHAZ), expressed in arbitrary units of $2^{-\Delta Ct}$. Two-way ANOVA was performed for comparison between the conditions. **A**. Wild-type. **B**. MCAD-KO 4. **C**. MCAD-KO 9. **D**. MCAD-KO 21. *** $p < 0.005$.

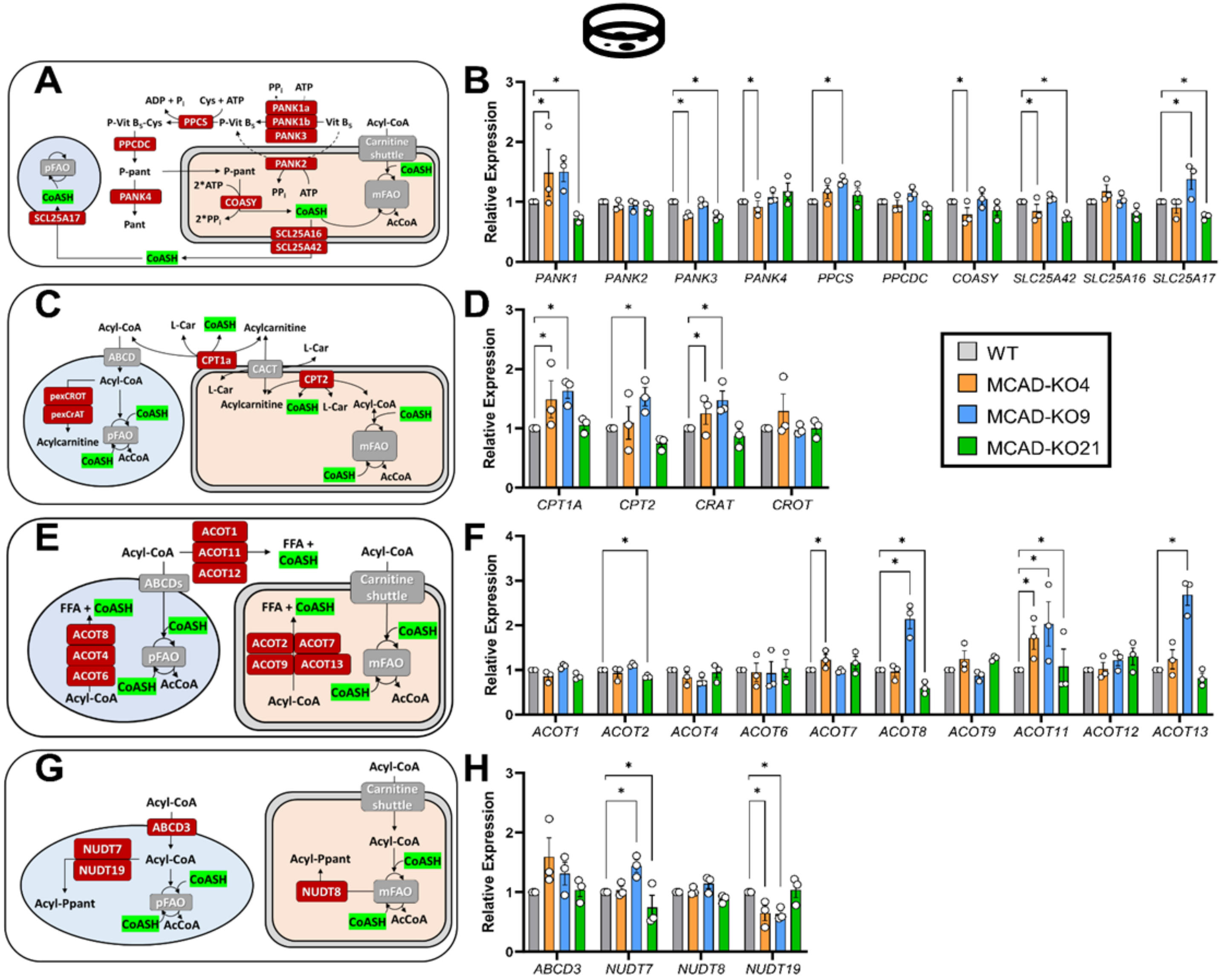

A
B
C
D
E
F
G
H
Relative Expression
PANK1
PANK2
PANK3
PANK4
PPCS
PPCDC
COASY
SLC25A42
SLC25A16
SLC25A17
CPT1A
CPT2
CRAT
CROT
ACOT1
ACOT2
ACOT4
ACOT6
ACOT7
ACOT8
ACOT9
ACOT11
ACOT12
ACOT13
ABCD3
NUDT7
NUDT8
NUDT19
WT
MCAD-KO4
MCAD-KO9
MCAD-KO21
PANK1a
PANK1b
PANK3
PANK2
PANK4
PPCS
PPCDC
COASY
SCL25A16
SCL25A42
SCL25A17
CoASH
pFAO
mFAO
Carnitine shuttle
Acyl-CoA
AcCoA
P-pant
Pant
ABCD
CPT1a
CACT
CPT2
pexCROT
pexCrAT
Acylcarnitine
L-Car
ACOT1
ACOT11
ACOT12
ABCDs
FFA + CoASH
ACOT8
ACOT4
ACOT6
ACOT2
ACOT7
ACOT9
ACOT13
ABCD3
NUDT7
NUDT19
NUDT8
Acyl-Ppant

Figure S11. Gene expression data from MCAD-KO HepG2 cells on *Palmitate no-glucose medium*. Relative expression of genes (relative to WT) in various pathways of CoA metabolism. Schemes of the relevant reactions are provided for clarity with three compartments: cytosol in white, mitochondria in pink, and peroxisomes in light bue. AcCoA = acetyl-CoA. **A & B.** CoA biosynthesis and transport. *PANK* = pantothenate kinase, *PPCS* = phosphopantothenate-cysteine ligase, *PPCDC* = phosphopantothenoylcysteine decarboxylase, *COASY* = bifunctional coenzyme A synthase, Vit B_5 = pantothenate, P-Vit B_5 - = phosphopantothenate, P-Vit B_5-Cys = phosphopantothenoyl-cysteine, P-pant = phosphopantetheine, Pant = pantetheine, *SLC25A* = solute carrier family protein 25A. **C & D.** Carnitine acyltransferases. *CRAT* = carnitine acetyltransferase, *CROT* = peroxisomal carnitine octanoyltransferase. **E & F.** *ACOT* = Acyl-CoA thioesterases. **G & H.** *ABCD3* = ATP-binding cassette domain protein 3, *NUDT* = nudix hydrolase. Expression values are relative to control WT (= 1); each data point represents the average of an independent experiment (3 experiments), which consisted of 4-5 technical replicates (cell cultures); ± standard error of the mean (SEM). **Statistics are displayed in Figure S12**.

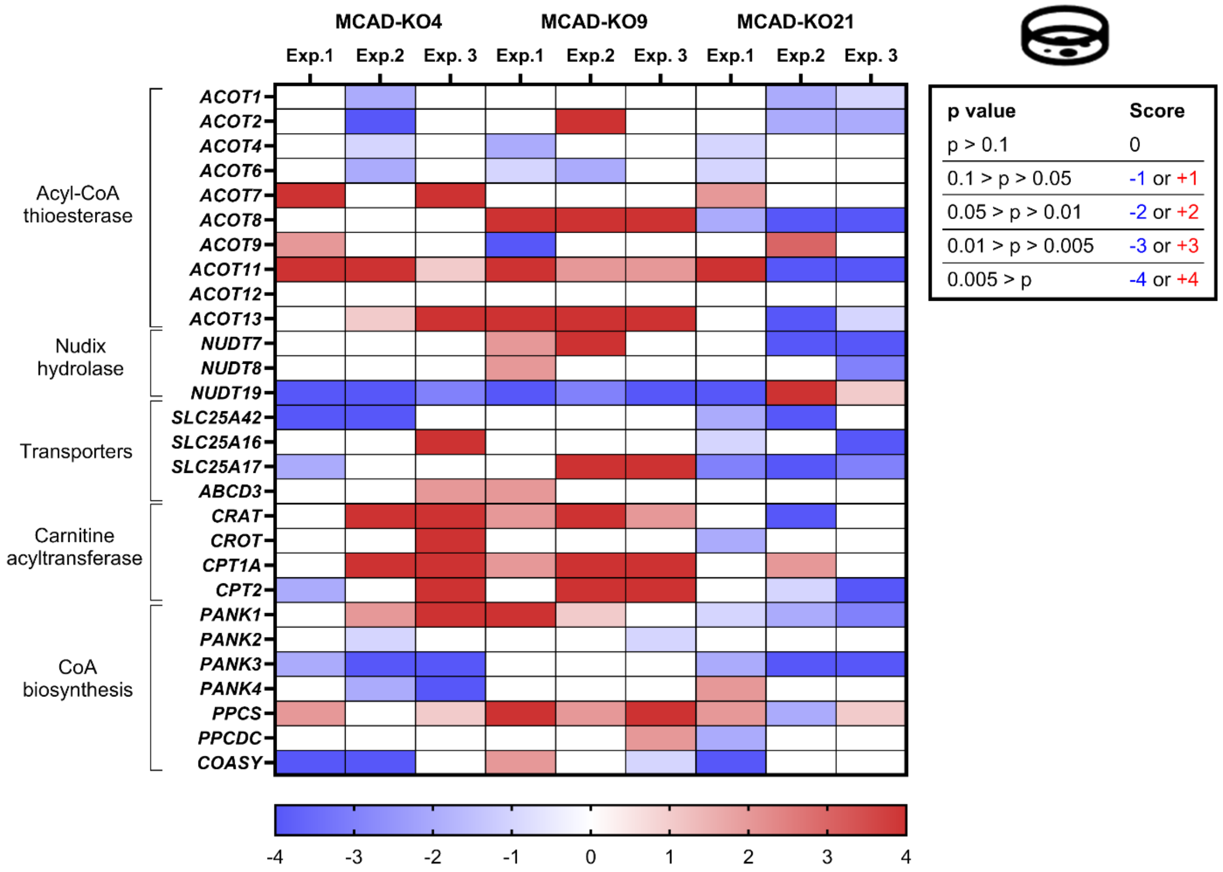

Figure S12. Heat map of gene expression data showing differences between MCAD-KO clones and WT incubated in *Palmitate no-glucose* medium. One-way Brown-Forsythe ANOVA adjusted by Dunnett's T3 multiple comparisons test was performed per individual experiment (n=3) on the data displayed in Fig. S11. These were therefore values relative to the WT mean within each experiment. Each experiment consisted of 4-5 cultures. The scores (-4 to +4) in the heat maps represent the p values as follow, 0: $p > 0.1$, +1 or -1: $0.1 > p > 0.05$, +2 or -2: $0.05 > p > 0.01$, +3 or -3: $0.01 > p > 0.005$, +4 or -4: $p < 0.005$. Red cells (and positive scores) indicate cases where the KO presented a gene upregulation relative to the WT, while blue cells (negative scores) indicate a gene downregulation relative to the WT. White cells indicate no significant changes (p>0.1).

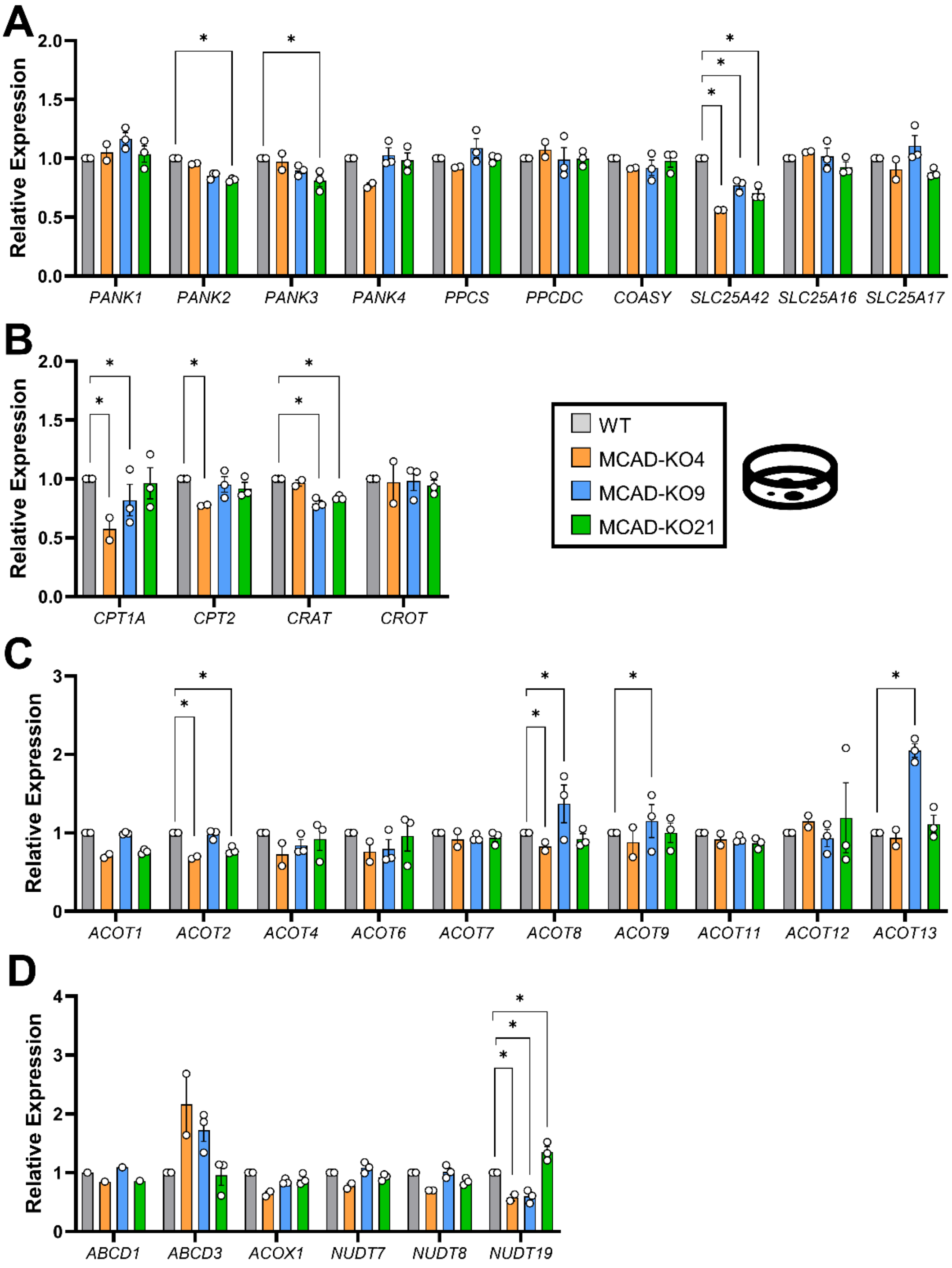

Figure S13. Gene expression data from MCAD-KO HepG2 cells on *Palmitate low-glucose* medium. Relative expression of genes (relative to WT) in various pathways of CoA metabolism. **A .** CoA biosynthesis and transport. *PANK* = pantothenate kinase, *PPCS* = phosphopantothenate-cysteine ligase, *PPCDC* = phosphopantothenoylcysteine decarboxylase, *COASY* = bifunctional coenzyme A synthase, *SLC25A* = solute carrier family protein 25A. **B.** Carnitine acyltransferases. *CRAT* = carnitine acetyltransferase, *CROT* = peroxisomal carnitine octanoyltransferase. **C.** *ACOT* = Acyl-CoA thioesterases. **D.** *ABCD* = ATP-binding cassette domain protein, *NUDT* = nudix hydrolase. Expression values are relative to control WT (= 1); each data point represents the average of an independent experiment (3 experiments), which consisted of 4-5 technical replicates (cell cultures); ± standard error of the mean (SEM). **Statistics are displayed in Figure S14**.

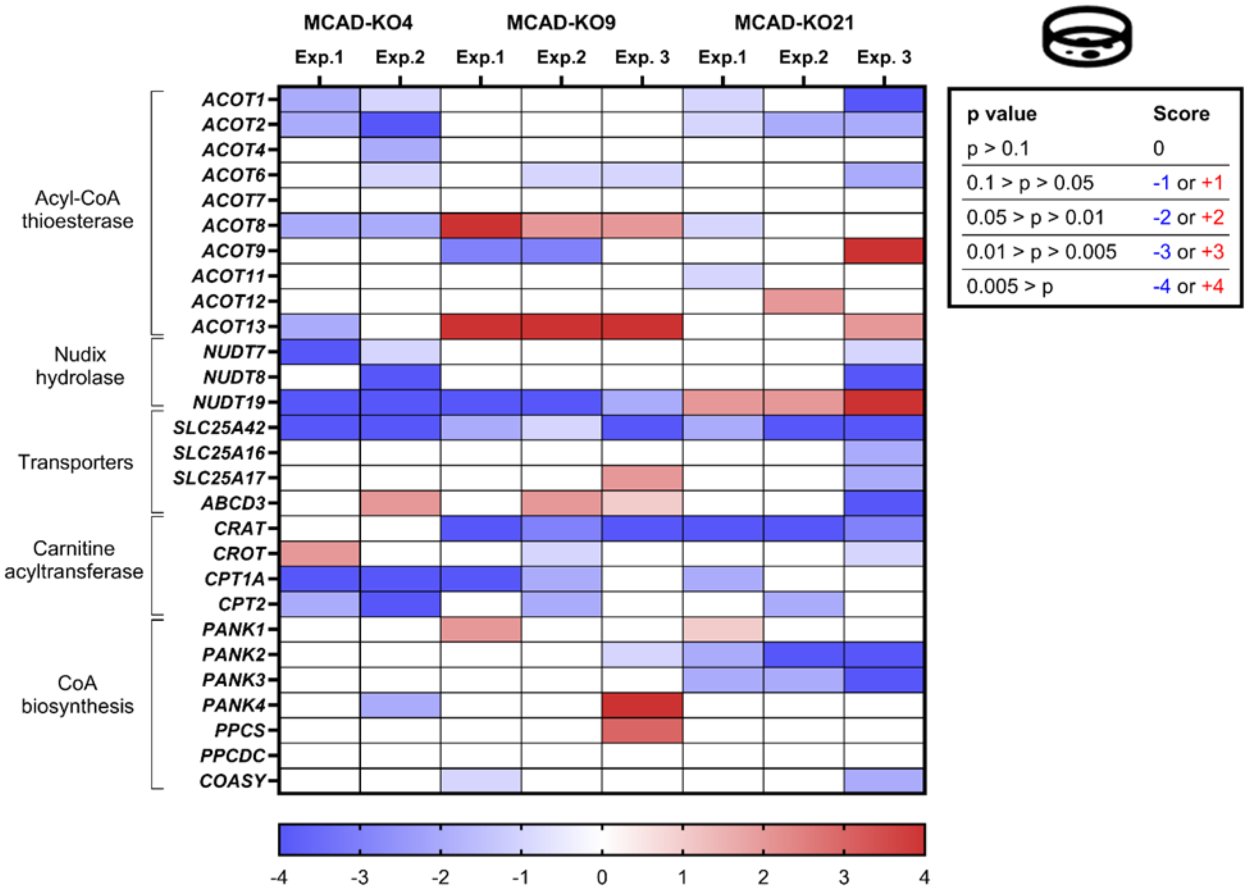

Figure S14. Heat map of gene expression data showing differences between MCAD-KO clones and WT incubated in *Palmitate low-glucose* medium. One-way Brown-Forsythe ANOVA adjusted by Dunnett's T3 multiple comparisons test was performed per individual experiment (n=3) on the data displayed in Fig. S13. These were therefore values relative to the WT mean within each experiment. Each experiment consisted of 4-5 cultures. The scores (-4 to +4) in the heat maps represent the p values as follow, 0: $p > 0.1$, +1 or -1: $0.1 > p > 0.05$, +2 or -2: $0.05 > p > 0.01$, +3 or -3: $0.01 > p > 0.005$, +4 or -4: $p < 0.005$. Red cells (and positive scores) indicate cases where the KO presented a gene upregulation relative to the WT, while blue cells (negative scores) indicate a gene downregulation relative to the WT. White cells indicate no significant changes (p>0.1).

Table S1. Statistical significance of differences in CoASH and total CoA levels between HepG2 cells divided in T0H and T24 groups. Two-way ANOVA adjusted by Sídák's multiple comparisons test was performed per individual experiment (n=3) on the data displayed in Fig. 3B-C. These were therefore values relative to the T0H mean within each experiment. Each experiment consisted of 4-5 cultures. Differences were considered statistically significant when $p < 0.05$. Red cells indicate cases where T24H had a higher concentration relative to the T0H, while blue cells indicate a lower concentration than T0H. Black cells indicate unavailable data.

	**WT**			**MCAD-KO4**			**MCAD-KO9**			**MCAD-KO21**		
**CoA species**	**EXP.1**	**EXP.2**	**EXP.3**	**EXP.1**	**EXP.2**	**EXP.3**	**EXP.1**	**EXP.2**	**EXP.3**	**EXP.1**	**EXP.2**	**EXP.3**
CoASH	0.787	0.588	0.783		0.469	0.492		0.969	0.956	0.454	0.999	0.287
Total CoA	0.022	0.611	0.879		0.023	0.244	0.0005	0.999	0.772	0.002	0.0003	0.100

Table S2. Statistical significance of differences in CoASH and total CoA levels between MCAD-KO clones and WT in T0H and T24H. One-way Brown-Forsythe ANOVA adjusted by Dunnett's T3 multiple comparisons test was performed per individual experiment (n=3) on the data displayed in Fig. 3D-E. These were therefore values relative to the WT mean within each experiment. Each experiment consisted of 4-5 cultures. Differences were considered statistically significant when $p < 0.05$. Red cells indicate cases where MCAD-KO had a higher concentration relative to the WT, while blue cells indicate a lower concentration than WT. Black cells indicate unavailable data.

MCAD-KO clones	MCAD-KO4			MCAD-KO9			MCAD-KO21		
**CoA species**	**EXP.1**	**EXP.2**	**EXP.3**	**EXP.1**	**EXP.2**	**EXP.3**	**EXP.1**	**EXP.2**	**EXP.3**
CoASH, 0H		0.254	0.082		0.020	0.177	0.024	0.004	0.935
CoASH, 24H		0.349	0.338	0.150	0.052	0.322	0.150	0.043	0.412
Total CoA, 0H		0.154	0.144	0.015	0.375	0.024	0.550	0.007	0.170
Total CoA, 24H		0.999	0.230	0.007	0.999	0.061	0.315	0.994	0.918

Table S3. Statistical significance of differences in percentage label incorporation into the CoASH fraction and into the total CoA pool over the course of 24 hours between T0H and T24 groups. Two-way ANOVA adjusted by Sídák's multiple comparisons test was performed per individual experiment (n=3) on the data displayed in Fig. 3F-G. These were therefore values relative to the T0H mean within each experiment. All groups passed on the Brown-Forsythe test, thus they did not present significant differences between their standard deviations. Each experiment consisted of 4-5 cultures. Differences were considered statistically significant when $p < 0.05$.

	WT			MCAD-KO4			MCAD-KO9			MCAD-KO21		
**CoA species**	**EXP.1**	**EXP.2**	**EXP.3**	**EXP.1**	**EXP.2**	**EXP.3**	**EXP.1**	**EXP.2**	**EXP.3**	**EXP.1**	**EXP.2**	**EXP.3**
CoASH	0.005	<0.0001	<0.0001		<0.0001	<0.0001	<0.0001	<0.0001	<0.0001	<0.0001	<0.0001	<0.0001
Total CoA	<0.0001	<0.0001	<0.0001		<0.0001	<0.0001	<0.0001	<0.0001	<0.0001	<0.0001	<0.0001	<0.0001

Table S4. Statistical significance of differences in percentage label incorporation into the CoASH fraction and into the total CoA pool over the course of 24 hours between MCAD-KO clones and WT. Two-way ANOVA adjusted by Sídák's multiple comparisons test was performed per individual experiment (n=3) on the data displayed in Fig. 3F-G. These were therefore values relative to the WT mean within each experiment. All groups passed on the Brown-Forsythe test, thus they did not present significant differences between their standard deviations. Each experiment consisted of 4-5 cultures. Differences were considered statistically significant when $p < 0.05$. Red cells indicate cases where MCAD-KO had a higher percentage label incorporation relative to the WT, while blue cells indicate a lower percentage than WT. Black cells indicate unavailable data.

**MCAD-KO clones**	**MCAD-KO4**			**MCAD-KO9**			**MCAD-KO21**		
**CoA species**	**EXP.1**	**EXP.2**	**EXP.3**	**EXP.1**	**EXP.2**	**EXP.3**	**EXP.1**	**EXP.2**	**EXP.3**
CoASH, 24H		0.1659	0.3086	0.0044	0.3116	0.0136	0.0418	0.9975	0.2229
Total CoA, 24H		0.9346	<0,0001	0.0456	0.0075	0.0282	0.3495	0.6915	0.0001

Table S5. List of human primer sequences used in RT-qPCR.

Gene Name	Forward and reverse primer sequence (5'- 3')
PANK1	Fwd: AGGTGTCAGCATTCTAGCCGTG<br>Rev: GGTCTCACAACCAGTCAGCAAG
PANK2	Fwd: CGTGGAGATAGCACCAAAGTGG<br>Rev: CAGGTCCTCTTTACTGACAGCC
PANK3	Fwd: TTGCCAGGTTGGGCTGTAGCAT<br>Rev: GCACACATTCGTGCCACAGAAC
PANK4	Fwd: TCGTGGATTCCTACAGCGAGTG<br>Rev: CTGTCCCTCTAAGGAGTAGCTC
PPCS	Fwd: TCCTGGCAGTAGAGTTCACCAC<br>Rev: GGCATTTCAGAGACAGGAACATAG
PPCDC	Fwd: CAAGAAGCTGGTGTGCGGAGAT<br>Rev: GTCAACTCTGCTGGAAGCCACT
COASY	Fwd: TGAGGTGTGGACTGCTGTCATC<br>Rev: TGGCTCTGTTCCACAAGCTGCT
SLC25A42	Fwd: AGTTCAGCGCACACGAGGAGTA<br>Rev: GTAGGTCAGTGAAGCGGCTGTC
SLC25A16	Fwd: ATGCTCCTACCCTTCTTGGCAG<br>Rev: TTGCATTCGCCGACGAGTCACA
SLC25A17	Fwd: GGTGGTAAACACCAGACTGAAGC<br>Rev: AGCCGAGATTCCTTCATCGCGA
CPT1b	Fwd: TGTATCGCCGTAAACTGGACCG<br>Rev: TGTCTGAGAGGTGCTGTAGCAC
CPT2	Fwd: GCAGATGATGGTTGAGTGCTCC<br>Rev: AGATGCCGCAGAGCAAACAAGTG
CRAT	Fwd: CCTACAGACCAACAAGGAGCCT<br>Rev: TGCATCTAGGCACACGGTGAAG
CROT	Fwd: CTAGTGAGGAGCGAACTCGATG<br>Rev: CCTCTGGTGTTACATGTGGACTG
ACOT1	Fwd: GGGTTTTGCTGTGATGGCTCTG<br>Rev: CAGCCCAACTCCTGGACCTTTT
ACOT2	Fwd: ATGGAGACGCTCCATCTGGAGT<br>Rev: GTGATGCCCTTCAGGAAAGAGG
ACOT4	Fwd: CTTTGCCACGTTGGCTCTAGCT<br>Rev: CCTAGAGAAATGCCCAAAAGCCC
ACOT6	Fwd: GAGCAATCCACTGGAGGAACAC<br>Rev: GAGCTTGTAGCCTTTCAGAGGC
ACOT7	Fwd: CTACACCTCCAAGCACTCTGTG<br>Rev: CCTTGTCCACATTCTTCAGCGAC
ACOT8	Fwd: GCTGACCACTGGATGCTCTATG<br>Rev: AGGTCACAGCTAGGACTCCATC
ACOT9	Fwd: ATCCACTCCGCCAAGATGTCTC<br>Rev: GATGTCTTCCCGACCCAGCTAA
ACOT11	Fwd: TCTGGTGCTCAAAGCCATCGTG<br>Rev: TCATCTGCGTCCAGGACCACAA
ACOT12	Fwd: GGAGGTTACCAGCACTGTGGAA<br>Rev: GCCAAATGTGCTGGACTTCCCA
ACOT13	Fwd: CGGAGTCAGTGTCGATATGAACA<br>Rev: GCCTTGTTGGTCAGATCCACAG
ABCD3	Fwd: GTTCCTTTAGCAACGCCAAATGG<br>Rev: CTCTTTCCGCAGCCATTTGGAC
NUDT7	Fwd: CTCCGTCCTTTTGCCATTGGTG<br>Rev: TGTCTGTAGGGTCACGCTTACC
NUDT8	Fwd: CTGGCAGTGCCCGAGGAGCA<br>Rev: GCCTACACCAGCAAGCACTGG
NUDT19	Fwd: GCACCACTCGCCGCTTTGACA<br>Rev: GTTGCCTCTGATGGAGATGACC
YWHAZ	Fwd: ACCGTTACTTGGCTGAGGTTGC<br>Rev: CCCAGTCTGATAGGATGTGTTGG

Fwd, forward primer; Rev, reverse primer.

Table S6. List of murine primer sequences used in RT-qPCR.

Gene Name	Forward and reverse primer sequence (5'- 3')
Pank1a	Fwd: GTTCGCCCAGCATGATTCTC<br>Rev: CTTAACCAGGGTTCCACCGAT
Pank 1b	Fwd: CTGAGCCTAACTCCATTCAACT<br>Rev: TCCACCGATATCCATACCAAAC
Pank 2	Fwd: TTGGGCATACGTGGAGCTTT<br>Rev: TCTCACATACATTTCAACAGGACAAG
Pank 3	Fwd: TCACTGGGAACCAAAGGATAAA<br>Rev: CAGTGAGGGATACTTCCCATTATAG
Pank 4	Fwd: GAAAACGGCCTCGCACTAAA<br>Rev: CGCTCTTCCCAGCCTAGTGA
Ppcs	Fwd: CTCTCAGTCCATTAGGCTCTTC<br>Rev: GGATCTTGTGTTCAGGCATTTC
Ppcdc	Fwd: TAACAACAGAGAGAGCCAAACA<br>Rev: GCTTCCACATCTCCCATTCA
Coasy	Fwd: GGAGGCCTTTGGAACAGATATT<br>Rev: GAGGATCTTCATCTGCTTCTTGT
Cpt1a	Fwd: CCATCCTGTCCTGACAAGGTTTAG<br>Rev: CCTCACTTCTGTTACAGCTAGCAC
Cpt1b	Fwd: GCACACCAGGCAGTAGCTTT<br>Rev: CAGGAGTTGATTCCAGACAGGTA
Cpt2	Fwd: CAACTCGTATACCCAAACCCAGTC<br>Rev: GTTCCCATCTTGATCGAGGACATC
Crat	Fwd: GCTGCCAGAACCGTGGTAAA<br>Rev: CCTTGAGGTAATAGTCCAGGGA
Abcd1	Fwd: GCTGTGACCTCCTACACTCTCC<br>Rev: AGTAGTGCCAGTTCCACCTCA
Abcd2	Fwd: GAACTACCCCTCAGCGACAC<br>Rev: ATGGCCTCTGTGGAATATAGAAC
Acot1	Fwd: AACATCACCTTTGGAGGGGAG<br>Rev: TCCCCAACCTCCAAACCATCA
Acot2	Fwd: AGTCAACGACGCAAAATGGTG<br>Rev: GCTCTTCCAATCCTGTTGGC
Acot3	Fwd: GCTCAGTCACCCTCAGGTAA<br>Rev: AAGTTTCCGCCGATGTTGGA
Acot4	Fwd: ACATCCAAAGGTAAAAGGCCCA<br>Rev: TCCACTGAATGCAGAGCCATT
Acot6	Fwd: ATCCTCAGGTGAAAGGCCCAA<br>Rev: AAGGACAGTGGCTGTGATGTT
Acot7	Fwd: ATCAGCACGCGGCACTGTAA<br>Rev: TTGGTACCTGTGAGGATGTTCTCC
Acot8	Fwd: AAGTATCGAGTGGGGCTGAAC<br>Rev: TGATGTCACCTTCCCCAATGT
Acot9	Fwd: GGGGCTTCTTACTCATGGCA<br>Rev: CATGGTCTCTCCAGACTGTGG
Acot11	Fwd: GTGACCAGCGGCCCTTTAG<br>Rev: AGAACATAGAGGCGAAGCCCCTT
Acot12	Fwd: CCGTGGCACTAAGGTCAGTT<br>Rev: ACGTTACGGTGCACGAATTG
Acot13	Fwd: AGACTCTTGCTTTGCGTCCA<br>Rev: GACAAGCGTCACCTTTTCCAA
Nudt7	Fwd: CCAAGTGGAGGTGGTCTCTC<br>Rev: GATGAAATCACGGCCAGACT
Nudt8	Fwd: CAGTTTCCCAGGCGGTAAGT<br>Rev: CACGTTGGCAAGTACTGGGA
Nudt19	Fwd: ATCTGTGCCATCCGCGAAGC<br>Rev: CACAGCTGGAGGAAGCAGCG
36b4	Fwd: GGACCCGAGAAGACCTCCTT<br>Rev: GCACATCACTCAGAATTTCAATGG

Fwd, forward primer; Rev, reverse primer.
